# Anchoring Atomically Precise Chiral Bismuth Oxido Nanoclusters on Gold: The Role of Amino Acid Linkers

**DOI:** 10.1021/acs.langmuir.4c01445

**Published:** 2024-07-12

**Authors:** Annika Morgenstern, Rico Thomas, Oleksandr Selyshchev, Marcus Weber, Christoph Tegenkamp, Dietrich R. T. Zahn, Michael Mehring, Georgeta Salvan

**Affiliations:** † Faculty of Natural Science, Institute of Physics, Semiconductor Physics, Chemnitz University of Technology, Chemnitz 09107, Germany; ‡ Faculty of Natural Science, Institute of Chemistry, Coordination Chemistry, Chemnitz University of Technology, Chemnitz 09107, Germany; § Faculty of Natural Science, Institute of Physics, Analysis of Solid Surfaces, Chemnitz University of Technology, Chemnitz 09107, Germany; ∥ Center of Materials, Architectures and Integration of Nanomembranes, Chemnitz University of Technology, Chemnitz 09126, Germany

## Abstract

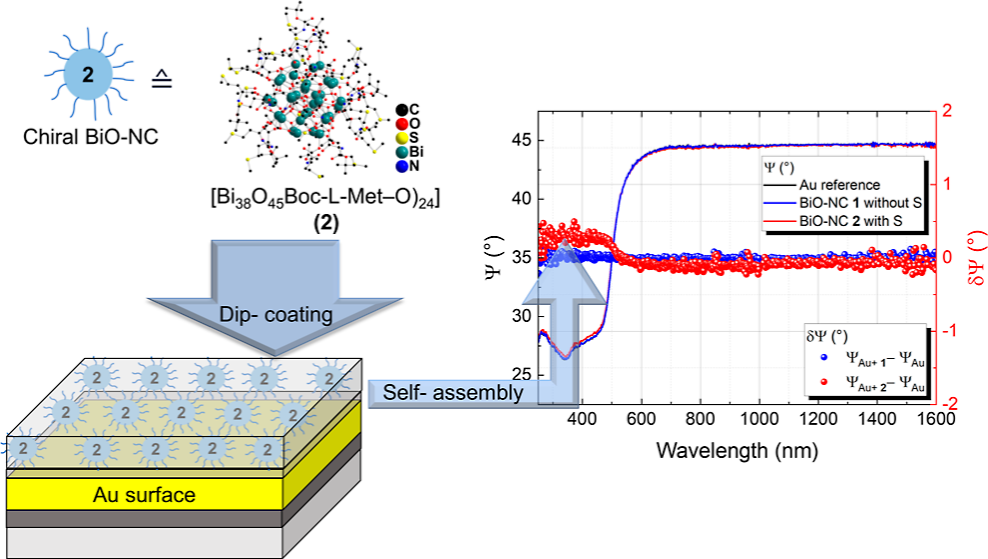

The adsorption of chiral molecules onto metallic surfaces triggers electron spin polarization at the interface, paving the way for applications in chiral opto-spintronics. However, the spin effects sensitively depend on the binding and ordering of the chiral species on surfaces. This study explores the adsorption of chiral thioether-functionalized atomically precise bismuth oxido nanoclusters (BiO-NCs) on gold (Au) surfaces, extending the conventional approach of using thiol-containing molecules and complexes to nanoclusters. Starting from the precursor [Bi_38_O_45_(NO_3_)_20_(dmso)_28_](NO_3_)_4_·4dmso (A), chiral BiO-NCs were synthesized by substituting the nitrates with *N*-(*tert*-butoxycarbonyl)-l-methionine (Boc-l-Met-O^–^) ligands to obtain [Bi_38_O_45_(Boc-l-Met-O)_24_] (**2**). The full exchange of nitrate by the Boc-l-methionine ligand was demonstrated by powder X-ray diffractograms, dynamic light scattering, electrospray ionization mass spectrometry, nuclear magnetic resonance, infrared, circular dichroism, and X-ray photoelectron spectroscopy. Compared to previously reported [Bi_38_O_45_(Boc-l-Phe-O)_24_(dmso)_9_] (**1**), BiO-NC **2** shows differences in the growth mode on a Au surface as revealed by scanning electron microscopy, wherefore a stronger binding of BiO-NC **2** is assumed. Anchoring of BiO-NC **2** to the Au surface through thioether groups induced a discernible change in the optical response of the Au surface analyzed by spectroscopic ellipsometry (SE). From the numerical modeling of the SE parameters, a layer thickness of ∼2 nm, corresponding to a monolayer of BiO-NC **2**, was estimated for the samples prepared by dip coating. Thus, strong adsorption of BiO-NC **2** to the Au surface is concluded, which is an essential prerequisite for chiral-induced interface spin polarization.

## 1 Introduction

The self-assembly of monolayers^1−3^ (SAMs) is well established for thiol-containing molecules, which are able to chemisorb to metallic surfaces in a highly ordered manner.^4−6^ In the context of the recent emerging field of chiral-induced spin selectivity (CISS),^7^ it was discovered that thiol anchoring is well suited to bind CISS molecules on metallic surfaces.^4,5^ So far, mainly molecules of helical chirality were studied for their CISS effects, although the concept is believed to be more general with regard to chiral species. In addition to the normal CISS effect, the combination of semiconductor nanoparticles as light absorbers and chiral ligands paved the way toward the photoinduced spin selectivity effect.^8,9^ Two approaches are attractive: (i) the light-absorbing system itself is chiral or (ii) the nonchiral light-absorbing system is connected through a helical molecule to the metallic substrate. The second approach was already successfully demonstrated using CdSe semiconductor quantum dots connected to helical polypeptides^8,9^ and for porphyrin-containing chiral scaffolds^10^ on metallic surfaces. Another approach might be realized using chiral atomically precise metal oxido nanoclusters (MO-NCs) as chiral light absorbers. Especially, we are interested in bismuth-based compounds because this element shows a large pool of accessible MO-NCs, with the option of their targeted optical absorption tuning via a recently developed doping procedure.^11−15^ In addition, the environmentally benign nature of bismuth compounds offers access to novel sustainable materials and medical applications and led already to developments with potential application, e.g., in catalysis, medicine, and electronics.^16−22^ Among the bismuth oxido nanoclusters (BiO-NCs), those with a [Bi_38_O_45_]^24+^ core seem to be the most stable ones, as evidenced by studies on various accessible BiO-NCs of this structure type covered with different ligands such as sulfonates, carboxylates, and nitrate.^23−28^ Furthermore, their physical properties such as the optical band gap can be tuned by doping the cluster core for example with rare earth elements.^12^ However, many of the BiO-NCs suffer from either low hydrolytic stability at the ligand periphery, low solubility, low biocompatibility of their ligands, or accessibility on a large scale.^11,15^ Recently, we were able to prepare a rather stable and highly soluble amino acid-modified BiO-NC of the type [Bi_38_O_45_(Boc-l-Phe-O)_24_(dmso)_9_] (**1**) and studied its deposition behavior using different coating techniques on gold surfaces. Our approach led to microstructured cluster agglomerations on the metal surface due to crystallization processes and strong intermolecular interactions between the clusters.^13^ To overcome this challenge, we synthesized the *N*-(*tert*-butoxycarbonyl)-l-methionine (Boc-l-Met-OH)-substituted BiO-NC [Bi_38_O_45_(Boc-l-Met-O)_24_] (**2**), which is expected to provide higher affinity to the gold substrate due to its thioether functionality (−S–CH_3_). Such thioethers have been demonstrated to form SAMs in a similar manner to thiols.^29^ Here, we report on the adsorption of BiO-NCs on Au, a prerequisite to study the CISS effect in such systems. The adsorption behavior of BiO-NC **2** to the Au surfaces was studied by X-ray photoelectron spectroscopy (XPS) and scanning electron microscopy (SEM) first. We found that the tendency of agglomeration was decreased while the homogeneity of the self-assembled film was increased in comparison to BiO-NC **1**. Given the sensitivity of spectroscopic ellipsometry (SE) to the adsorbed material, we employed this technique to monitor changes in the adsorption of BiO-NCs. Specifically, we analyzed the difference spectra for the ellipsometric parameters Ψ and Δ,^30^ to define whether strong chemical interaction between the BiO-NCs and the Au surface occurs, which can be proven by a spectral feature present in the energy dispersion spectra of those parameters. Additionally, we successfully derived the dielectric function for BiO-NC **2** through a modification of the three-layer Arwin model.^31^

## 2 Materials and Methods

### 2.1 Materials

*N*-(*tert-*Butoxycarbonyl)-l-phenylalanine (99%), *N*-(*tert-*butoxycarbonyl)-l-methionine (98%), Na_2_CO_3_ (99.5%), and *N*-methyl-2-pyrrolidone (NMP) were obtained from Sigma-Aldrich and used without further purification. Bi(NO_3_)_3_·5H_2_O (98%) and spectroscopic grade ethanol (99.9%) purchased from Alfa Aesar were used without further purification. The synthesis of [Bi_38_O_45_(NO_3_)_20_(dmso)_28_](NO_3_)_4_·4dmso (**A**),^27^ Boc-l-Phe-ONa (**B**),^13^ and [Bi_38_O_45_(Boc-l-Phe-O)_24_(dmso)_9_] (**1**)^13^ was carried out according to the procedure described in the literature. The sodium salt of Boc-l-Met-OH (**C**) was synthesized in accordance with the synthesis procedure for compound **B**.^13^

### 2.2 Synthesis of [Bi_38_O_45_(Boc-l-Met-O)_24_] (**2**)

The BiO-NC [Bi_38_O_45_(NO_3_)_20_(dmso)_28_](NO_3_)_4_·4dmso (**A**, 1.67 g, 0.135 mmol) was added to 40 mL of dmso and the resulting dispersion was heated to 80 °C for 1 h to give a colorless solution. Boc-l-Met-ONa (**C**, 1.32 mg, 4.87 mmol) was subsequently added and the colorless solution was stirred at 80 °C for 4 h. The hot solution was filtrated and allowed to cool to ambient temperature. A colorless solid of **2** was obtained after slow evaporation of the solvent for 3 weeks. The solid was washed with 15 mL of deionized water and then dried in vacuum at 60 °C for 2 h. Compound **2** was obtained as a colorless solid (1.478 g, 0.098 mmol, 72% based on the bismuth content in **A**).

^1^H NMR (ppm, 500.30 MHz, MeOD-*d*_4_, 298 K): δ 4.15 (s, 1H), 2.66 (s, dmso_coord_), 2.60 (m, 2H), 2.18 (m, 1H), 2.14 (s, 3H), 2.01 (m, 1H), 1.48 (s, 9H). ^13^C NMR (ppm, 125.80 MHz, MeOD-*d*_4_, 298 K): 180.7, 157.8, 80.5, 56.3, 33.6, 31.9, 29.2, 16.0. CHNS (%, expt and calcd) for Bi_38_O_141_C_240_H_432_S_24_N_24_ (M = 14620.94 g·mol^–1^): C, 19.13 (19.72); H, 3.02 (2.98); N, 2.48 (2.30); S, 5,02 (5.26). IR (cm^–1^): 3340 m, 2974 m, 2916 m, 1685 m, 1558 m, 1491 m, 1388 s, 1363 s, 1247 m, 1161 s, 1048 s, 1023 s, 955 m, 859 m, 776 m, 503 s.

### 2.3 Sample Preparation

The bismuth oxido clusters [Bi_38_O_45_(Boc-l-Phe-O)_24_(dmso)_9_] (**1**) and [Bi_38_O_45_(Boc-l-Met-O)_24_] (**2**), respectively, were dissolved in ethanol of spectroscopic grade. A mass concentration of 20 mg·ml^–1^ was used for all ex-situ experiments. The mixture was heated to 80 °C for 5–10 min to give a clear solution. Polycrystalline Au-coated (100 nm) Si/SiO_2_ substrates modified with a 20 nm adhesion layer of titanium were used. The samples were cleaned using NMP at 80 °C for 10 min, followed by a subsequent rinsing procedure using isopropanol, deionized water, and spectroscopic-grade ethanol. The cleaned substrates were stored in spectroscopic ethanol until use.^6^ The NMP-treated samples were dipped for 2 h in the cluster-containing solutions, while the vials were sealed and kept in nitrogen atmosphere. After the dipping procedure, the samples were rinsed by spectroscopic-grade ethanol to remove all unbound residuals and subsequently dried with a nitrogen stream. In the Results and Discussion section, we will not only focus on washed samples but also on unrinsed ones. Therefore, we mark measurements taken on the rinsed samples with (w). A description of the sample preparation method and assignment is given in Table S1.

## 3 Characterization Methods

SEM measurements were performed using a NanoNovaSEM200 (Thermo Fisher Scientific) device with an electron beam energy of 10 keV. The images shown in this work were detected at magnifications of 80,000× (with a TLD detector) and at 1200× (with an EDN detector). Powder samples were measured by attenuated total reflection Fourier transform infrared (ATR-FTIR) spectroscopy in the range of 450–4500 cm^–1^ using a *Nicolet iS 5 FT-IR* spectrometer (Thermo Fisher Scientific) with an iD7 AR-coated diamond crystal ATR accessory. Film samples were investigated by FTIR spectroscopy in the range of 450–4500 cm^–1^ using a VERTEX 80v FTIR spectrometer (Bruker) with an ATR unit. Powder X-ray diffractograms were measured at ambient temperature with a *STADI P* diffractometer (Stoe) using Ge(111)-monochromatized Cu–K_α_ radiation (1.54056 Å, 40 kV, 40 mA). Particle size distribution (PSD) based on dynamic light scattering (DLS) was determined using a Zetasizer Nano ZS (Malvern Instruments), allowing the characterization of dispersions and suspensions in a size range of 0.4 nm to 6 μm. A red laser (633 nm, 4 mW) was used as a light source, and the analyses were performed at an angle of 173° (noninvasive backscattering default). The compounds were dissolved in appropriate solvents (20 g·L^–1^), filtered [0.45 μm pore size, poly(tetrafluoroethylene)], filled into glass cuvettes (DTS0012), and measured at 20 °C. Calculation of the PSD was carried out according to the “Mie theory”, assuming the presence of spherical particles.^32,33^^1^H and ^13^C{^1^H} nuclear magnetic resonance (NMR) spectra were recorded at room temperature in MeOD-*d*_4_ (dried over 4 Å molecular sieve) with an *AVANCE III 500* spectrometer (Bruker) at 500.30 and 125.80 MHz, respectively, and were referenced internally to the deuterated solvent relative to Si(CH_3_)_4_ (δ = 0.00 ppm). Quantitative elemental analyses of the elements C, H, N, and S were carried out with a *varioMICRO cube* (Elementar Analysensysteme GmbH). Electrospray ionization mass spectrometry (ESI-MS) was carried out using a trapped ion mobility spectrometry time-of-flight mass spectrometer (Bruker Daltonik GmbH). Calibration was performed in the *m*/*z* range of 100–10000 using cesium perfluoroheptanoate (*c* = 5 mM in H_2_O/MeCN with V:V = 1:1, abcr GmbH). The crystals of compound **2** were dissolved in MeOH (Ultra LC-MS grade, Carl Roth) with a final concentration of 100 μM. The sample was injected into the ESI source using a Hamilton syringe (*V* = 500 μL) at a flow rate of 180 μL·h^–1^. The voltage of the spray capillary was set to 4.5 kV (positive mode) with a deflection and an end plate offset voltage of 70 and 500 V, respectively. The dry gas flow was about 3.0 L·min^–1^ and a drying temperature of 200 °C was used. Mass spectra were treated by baseline subtraction (flatness 0.3) and smoothing (Savitzky–Golay algorithm, width 0.04 *m*/*z*). Isotope patterns were calculated using Bruker Compass Data Analysis software (Copyright © 2023 Bruker Daltonik GmbH, version 6.1). XPS analysis was performed with an ESCALAB 250Xi photoelectron spectrometer (Thermo Fisher Scientific) in an ultrahigh vacuum (UHV) chamber using a monochromatic Al–K_α_ (1486.68 eV) X-ray source. The X-ray beam diameter was 900 μm. XPS survey spectra were acquired at a pass energy of 200 eV. High-resolution spectra were measured at a pass energy of 20.0 eV providing a spectral resolution of 0.5 eV. To prevent massive charging effects, all XPS spectra were acquired using a built-in charge compensation gun. The binding energies of films on gold were referenced to the binding energy of Au 4f_7/2_ (84.0 ± 0.1) eV. The binding energies of powders were referenced to the binding energy of Bi 4f_7/2_ (159.4 ± 0.1) eV. XPS data were analyzed using Avantage software; ALTHERMO1 (adjusted Scofield) sensitivity factors were used for quantification. To measure the variable angle spectroscopic ellipsometry, a M2000 ellipsometer (J.A. Woollam Co.) was used, with angles of incidence (AOIs) in the range of 45–75° in steps of 5° with an acquisition time of 10 s. The spectral range was chosen between 250 and 1600 nm (4.96–0.78 eV). For all measurements, focusing optics were employed proving a spot size of about 200 μm. The analysis of the recorded spectra was performed by modeling the data using the software CompleteEASE (J.A. Woollam Co.).^34^ The circular dichroism (CD) spectra were recorded with a CD spectrophotometer J-1500 (Jasco Deutschland GmbH) in 10 mm cuvettes for the range of 200–400 nm with a standard air-cooled 150 W xenon lamp as the light source. Conditions for the CD measurements were 50 nm·min^–1^ scanning speed, 1 nm bandwidth, 0.1 nm data pitch, and 4 s data acquisition time. For CD measurements, the compounds were dissolved in acetonitrile (high-performance liquid chromatography plus grade, 99.9 %, Sigma-Aldrich) in concentrations between 10^–3^ and 10^–6^ mol·l^–1^. UV–vis spectroscopy was performed using a *Cary 60 UV–vis* (Agilent Technologies) equipped with a *Barrelino* (Harrick Scientific Products) remote diffuse reflection probe.

## 4 Results and Discussion

### 4.1 Cluster Characterization

As recently reported, BiO-NCs with amino acids in the cluster periphery, such as [Bi_38_O_45_(Boc-l-Phe-O)_24_(dmso)_9_] (**1**), show an enhanced solubility in many organic solvents in contrast to other BiO-NCs such as [Bi_38_O_45_(NO_3_)_20_(dmso)_28_](NO_3_)_4_·4dmso (**A**). This allows the Boc-l-Phe-O^–^-protected BiO-NC **1** to be deposited on gold surfaces via different solvent-based deposition methods.^13^ However, the previously prepared films showed a rough surface and cluster agglomerations due to weak adsorption to the Au substrate, producing films unsuitable for SE measurements. Thus, the Boc-l-Met-O^–^-protected BiO-NC [Bi_38_O_45_(Boc-l-Met-O)_24_] (**2**) was synthesized to enhance the interaction between the Au surface and the cluster via sulfur–gold interaction similar to SAMs. In that way, the self-assembly of BiO-NC **2** was targeted via a dip coating process.

**Figure 1:**
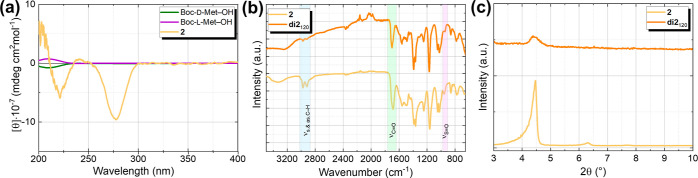
(a) CD spectra of Boc-d-methionine (*c* = 1 × 10^–3^ M), Boc-l-methionine (*c* = 1 × 10^–3^ M), and cluster **2** (*c* = 1 × 10^–5^ M) in acetonitrile. (b) IR spectra of BiO-NC film **di2**_120_ (top) and bulk of BiO-NC **2** (bottom). (c) PXRD pattern of BiO-NC **2** powder (bottom) and film **di2**_120_ (top).

The synthesis procedure of BiO-NC **2** in good yields (∼70%) is performed starting from [Bi_38_O_45_(NO_3_)_20_(dmso)_28_](NO_3_)_4_·4dmso (**A**) and the sodium salt of Boc-l-Met-OH (**C**) according to our previous work,^13^ which is outlined together with details of the material characterization in the Supporting Information. In order to investigate the chirality transfer from the amino acids to the BiO-NC, CD spectroscopy measurements were performed (cf. Figure 1a). Results for BiO-NC **1** are alike to those of former studies on a similar cluster compound and can be found in Figure S1a. For Boc-l-methionine and Boc-d-methionine, a Cotton effect signal at approximately 210 nm was observed as expected. BiO-NC **2** shows a broad Cotton effect signal between 250 and 300 nm with a maximum at 277 nm, which is attributed to the chiral signal of BiO-NC **2**. In addition, at around 210 nm, a chiral signal, which is close to that of Boc-l-methionine, was observed. These results are in line with those observed by CD spectroscopy of the cluster [Bi_38_O_45_(Boc-l-Val-O)_22_(OH)_2_].^35^ Noteworthily, the UV–vis absorption spectra of BiO-NC **2** show an absorption maximum around 280 nm (cf. Figure S2), comparable to the feature observed in its CD spectrum. We further investigated the in situ ligand exchange reaction of BiO-NC **2** with Boc-d-Met-OH using CD spectroscopy (Figure S1b).

Similarly, as previously shown for another valine-substituted BiO-NC,^35^ the ligand exchange was demonstrated by the change of the sign of the respective peak stemming from the Cotton effect (cf. Figure S1b). Hence, the chiral modification of the respective BiO-NCs by amino acid functionalization was proven. ESI-MS (cf. Figures S3 and S4 and Table S2) analysis as well as PXRD (cf. Figure S5), DLS (cf. Figure S6), and NMR (cf. Figure S7) measurements prove that the nanoclusters maintain their structure intact in the gas phase, solution, and solid state, respectively. This allows the BiO-NCs to be deposited on substrates using solvent-based methods while retaining the cluster structure.^13^ After the dipping process, selected BiO-NC films were rinsed with ethanol to remove nonbonded residuals from the surfaces, while other films were not treated with ethanol for comparison.

**Figure 2:**
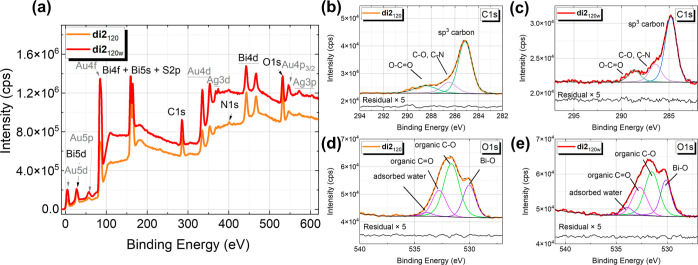
(a) XPS survey spectrum fragments for **di2**_120_ and **di2**_120w_ films. The peaks marked in gray arise from the substrate, while the black marked ones stem from the BiO-NCs. XPS high-resolution C 1s spectra with deconvolution for (b) **di2**_120_ and (c) **di2**_120w_ and O 1s spectra with deconvolution for (d) **di2**_120_ and (e) **di2**_120w_.

The resulting films were analyzed together with the bulk material of BiO-NC via IR spectroscopy, as well as XPS and PXRD. The comparison of the IR spectra of BiO-NC **2** with the corresponding starting materials Boc-l-Met-ONa (**C**, cf. Figure S8) and BiO-NC **A** (cf. Figure S9) is given in the Supporting Information. For detailed material characterization of BiO-NC **1** as well as for the corresponding dip-coated samples, we refer to our previous work.^13^ FTIR spectra of BiO-NC **2** and the dipped sample **di2**_120_ are depicted in Figure 1b. A comparison of the vibrational modes from a bulk sample BiO-NC **2** with those of a **di2**_120_ film shows similar spectra with minor shifts in the band positions. Hence, the molecular structure of BiO-NC **2** after the deposition is preserved in the **di2**_120_ film. In both spectra, the C–H valence vibrations ν_C–H_ at about 2974 and 2917 cm^–1^ and the amide C=O stretching vibration at around 1690 cm^–1^ are clearly observed.^13,36^ In addition, similarly strong vibrational modes in the fingerprint area were detected. All intensive peaks as well as the associated vibrations of the Boc-protected-l-methionine ligand are summarized in Table S3. Besides the peaks for the amino acid ligand, a minor amount of DMSO is indicated by the ν_S=O_ vibration at around 950 cm^–1^, which is in line with the results of NMR spectroscopy of BiO-NC **2** (cf. Figure S7).^13,36^ Unfortunately, the detection limit of the IR spectrometer does not allow the rinsed film **di2**_120w_ to be analyzed (cf. Figure S10). Thus, X-ray diffraction (XRD) was carried out on the **di2**_120_ and **di2**_120w_ films. Similar to the results obtained by IR spectroscopy, the typical XRD pattern for BiO-NC is observed solely for **di2**_120_. The resulting patterns of the films compared to that of bulk material of BiO-NC **2** are shown in Figure S11. The typical main diffraction for BiO-NC **2** at around 2θ = 4.46° (*d* = 1.98 nm), corresponding to the interlayer distance of a hexagonal closed packed cluster arrangement, is observed for **di2**_120_ and **2** (cf. Figure 1c). Finally, the chemical composition of the BiO-NC **2** powder (cf. Figure S12) as well as those of **di2**_120_ and **di2**_120w_ films (cf. Figure 2) was probed by XPS. The XPS determined that the elemental ratio of the powder **2** is in fair agreement with the calculated one (cf. Table S4), while the specific ratios of the films **di2**_120_ and **di2**_120w_ deviate from the calculated values of carbon, oxygen, and sulfur. The increased carbon and oxygen content in the films are most likely due to the adventitious carbon on the film samples.^37^ For powder BiO-NC **2**, a broadening of the XPS peaks (Figure S13a–f) due to uncompensated sample charging and a color change of the X-ray irradiated area most likely due to partial organic ligand degradation (cf. Figure S12) are observed. In addition, partial decomposition of the Boc-l-methionine ligands under XPS conditions is concluded resulting in an excess of oxygen from the nanocluster core and therefore in the deficiency of sulfur while nitrogen is only slightly affected in both films. This might be explained by radiation-induced damage at the −S–CH_3_ Au interface due to high secondary electron emission from the gold substrate.^38−41^ Major ligand loss due to hydrolysis during the preparation procedure is ruled out since both N and S content would be affected to the same extent. Figure 2a shows fragments of survey XPS spectra of the **di2**_120_ and **di2**_120w_ films in the binding energy range, where the signals of the BiO-NC **2** elements are expected. Indeed, in addition to the core level peaks of gold (Au 5d, Au 5p, Au 4d, and Au 4p_3/2_) and silver (Ag 3d and Ag 3p) originating from the substrate, the series of bismuth peaks (Bi 5d, Bi 4f, Bi 5s, and Bi 4d) as well as the O 1s, N 1s, and C 1s peaks were detected. The corresponding high-resolution C 1s and O 1s spectra are shown in Figure 2b–e and the spectra for N 1s are given in Figure S13d. The S 2p core level peak of sulfur, expected at around 160–164 eV, overlaps with the strong Bi 4f peaks manifesting themselves in the same binding energy range. Accordingly, the peak described in the literature for a thioether–gold bond at approximately 162 eV remains unresolved.^29^ The sulfur was probed by the S 2s core levels (Figure S13e), which are less common in XPS analysis due to the lower photoionization cross section as compared to the S 2p.^42^ The **di2**_120_ and **di2**_120w_ films revealed a pair of the S 2s peaks at ∼226 and ∼232 eV, which can be assigned to the S–Au (or C–S–C) bond and oxidized SO_*x*_ sulfur (e.g., DMSO residuals), respectively.^43^ However, the XPS spectrum of powder **2** demonstrates the S 2s peak at ∼228 eV, which is assigned to the C–S–C group of Boc-l-methionine (Figure S12e). The difference between the results for bulk material and film samples indicates a strong interaction of the sulfur atoms of BiO-NC **2** with gold and film damage at the −S–CH_3_ Au interface under XPS conditions as described before. Similar to the case of the BiO-NC **1** described by us previously,^13^ in the high-resolution N 1s spectra (cf. Figure S13d) for the **di2**_120_ and **di2**_120w_ films, only one peak at ∼400 eV is presented, which is assigned to the amide group of Boc-l-methionine. Any further peaks, such as the one characteristic for the nitrate cluster **A** at approximately 405.8 eV, are not detected.^13^ Hence, in correlation with the findings from the ESI-MS analysis (refer to Table S2 and Figure S3), it is substantiated that a comprehensive ligand exchange from nitrate to Boc-l-methionine in BiO-NC **2** occurred. In the O 1s high-resolution spectra (cf. Figure 2d,e), several chemical components can be revealed by deconvolution. The peak with the lowest binding energy at ∼530.1 eV corresponds to the Bi–O bond, which confirms the preservation of the bismuth oxido cluster core. The main peak with a binding energy of ∼531.6 eV can be assigned to the C–O bond, while the peak at ∼532.7 eV corresponds to the C=O bond, which both are present in the Boc-l-methionine ligand. A shoulder at ∼533.9 eV can be attributed to adsorbed water. It should be noted that partial hydrolysis of the BiO-NC (cf. Supporting Information Figure S4b,e), by which a minor amount of Boc-l-methionine ligands is replaced by OH during sample preparation, cannot be completely ruled out. The high-resolution C 1s spectra (cf. Figure 2b,c) show a dominant peak at 285.1 eV, which stems from the sp^3^-hybridized carbon predominantly present in the Boc group as well as l-methionine. In addition, high binding energy peaks recognized at ∼286.5 and ∼288.5 eV correspond to the C–O, C–N, and C=O groups of the ligand, respectively. In general, after rinsing the films with ethanol, the intensities of the peaks originating from the BiO-NCs **1** and **2** (e.g., Bi 4f) decrease, while the intensities of the substrate peak (e.g., Au 4f) increase (cf. Figure S13). This confirms that the washing process removes those parts of the BiO-NC layer, which are not bonded or physically adsorbed to the Au substrate. A comparison of the different XPS high-resolution spectra for the films **di1**_120_, **di2**_120_, **di1**_120w_, and **di2**_120w_ is shown in the Supporting Information (cf. Figure S13). It is important to note that the peaks for **di2**_120w_ are more intense compared to those of **di1**_120w_, while the intensity of the Au peaks behaves reversely. This shows that BiO-NC **2** has higher affinity to gold substrates, most likely as a result of the thioether anchor group as compared to BiO-NC **1**. Thus, the XPS results confirm the successful deposition of BiO-NC **1** and BiO-NC **2** on gold substrates, with higher affinity of BiO-NC **2** to gold. In the following part, the interactions as well as the resulting morphologies were further investigated.

### 4.2 Morphology and Film Thickness Analysis

**Figure 3:**
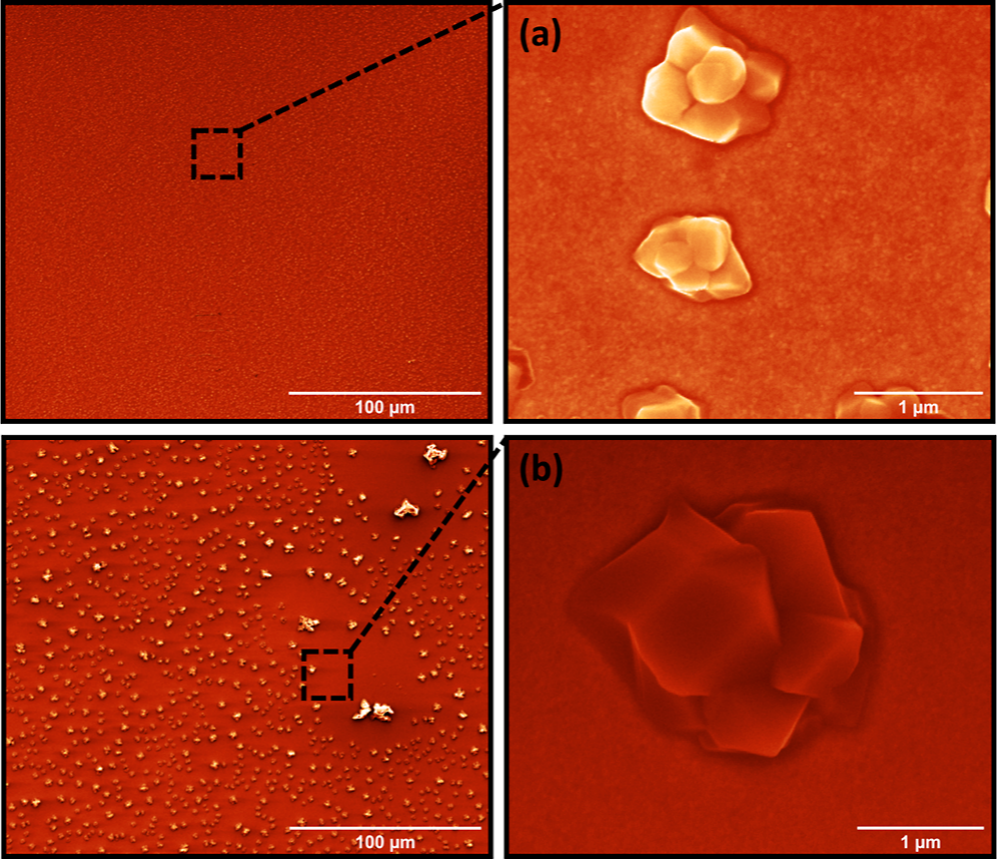
SEM images for (a) BiO-NC **2** (**di2**_120_) and (b) BiO-NC **1** (**di1**_120_) before washing the substrates by absolute ethanol. The distribution of BiO-NC **2** (a) is much more homogeneous, which results from the functionalization of the bismuth nanocluster by the amino acid Boc-l-methionine. In (b), significant cluster agglomeration by Ostwald ripening, as a thermodynamically driven process, was observed.

We recently reported on the growth behavior of BiO-NC **1** on Au and observed a tendency for agglomeration of the nanoclusters, coupled with a rather weak adsorption to the Au surface.^13^ In Figure 3a,b, the growth mode behavior of BiO-NC **1** (**di1**_120_) and **2** (**di2**_120_) is compared. The SEM images show that the BiO-NC **1** tends to agglomerate according to the Ostwald ripening process^44^ and form islands with well-defined terraces,^13^ while the agglomeration of BiO-NC **2** is much weaker. Only very small agglomerations can be distinguished, which are explained by a stronger interaction between the BiO-NC **2** and the Au surface via the sulfur atom present in the thioether group (−S–CH_3_). Therefore, by functionalizing the BiO-NCs, it becomes possible to manipulate the growth behavior and enhance the homogeneity and uniformity of the resulting film. This assertion is further supported by atomic force microscopy (cf. Figure S14 and associated Table S5). The agglomeration area decreases approximately nine times by changing the amino acid from Boc-l-phenylalanine to Boc-l-methionine in the cluster periphery. The maximum height is significantly decreased by five times, respectively. For comparison, the rinsed films **di1**_120w_ and **di2**_120w_ were analyzed using SEM (cf. Figure S15). However, the cluster agglomerates on the gold substrate were removed by the rinsing process resulting in a homogeneous surface for both **di1**_120w_ and **di2**_120w_ films. Since SEM resolution does not allow further analysis, XPS measurements were used to determine the film thickness.

Considering the exponential decay of the XPS signal with depth and that the inelastic mean free path of the photoelectrons (λ) depends on their kinetic energy (E), the film thickness can be estimated by the following equation^45^
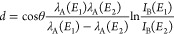
1where *d* is the thickness of the film “A”; θ is the photoelectron detection angle (cos θ = 1 for the normal angle); λ_A_(*E*_1_) and λ_A_(*E*_2_) are inelastic mean free paths of electrons in the material “A” at the kinetic energies *E*_1_ and *E*_2_, respectively; and *I*_B_(*E*_1_) and *I*_B_(*E*_2_) are intensities of two different core-level peaks registered from the substrate “B”. The method is simplified and assumes only inelastic scattering and ideally flat and homogeneous films. It is also limited for films, which are thin enough to transmit photoelectrons from the substrate. Additionally, the accuracy of the method can be significantly increased if the core levels used for the thickness estimation are separated by at least 300 eV.^45^

**Figure 4:**
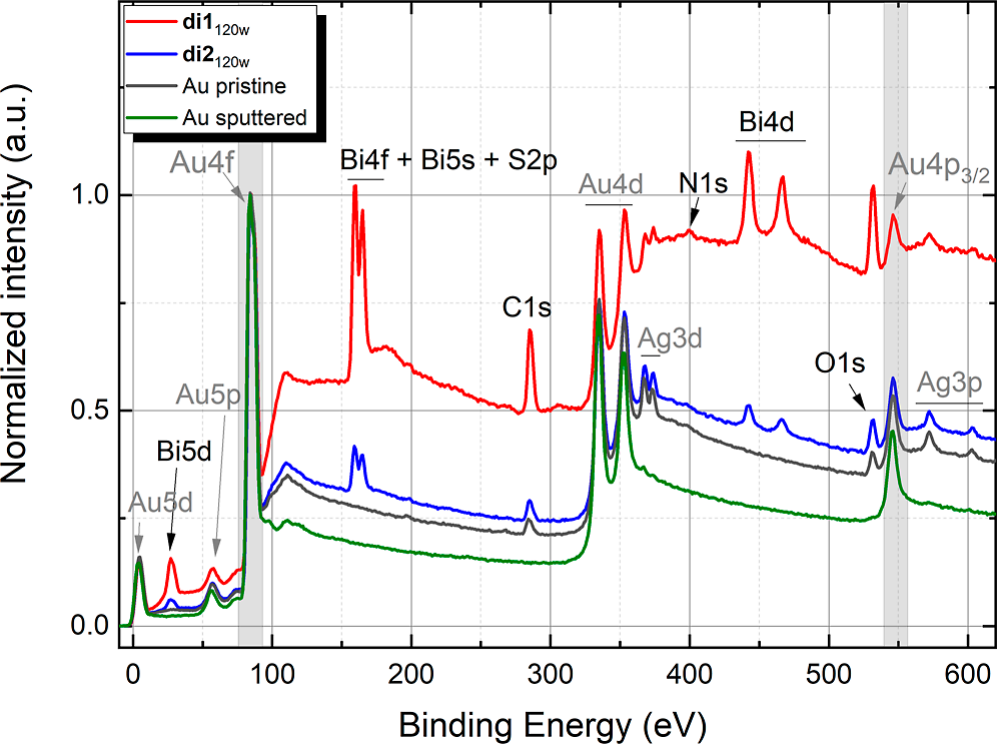
XPS survey spectrum fragments utilized to estimate the film thickness using core-level peaks from the Au substrates (peaks corresponding to the substrates are marked in gray; gray boxes indicate the peaks used for the calculations). Furthermore, the Au substrate was in situ cleaned by Ar^+^ sputtering in UHV and is shown for comparison. Note that the larger thickness of the **di2**_120w_ film can also be judged from the signal of higher intensity of BiO elements—Bi 4f and O 1s.

The thicknesses of the rinsed films of the BiO-NCs **1** and **2** with Boc-l-phenylalanine and Boc-l-methionine ligands were estimated from the XPS spectra (cf. the spectra for pristine Au substrates with adventitious carbon can be found in Figure 4) using normalized areas of the core-level peaks of the Au substrate. Note that the experimental peak areas already account for the transmission function of the spectrometer and the core-level sensitivity factors. The pair of Au 4f and Au 4p_3/2_ core levels was chosen for the calculations due to a significant difference in their kinetic energies. The values of the inelastic mean free path of electrons in the BiO-NCs were obtained using the Tanuma–Powell–Penn method.^46^ The band gap of BiO-NC **2** was determined to be *E*_g_ = 3.5 eV (cf. Tauc plot in Figure S2b). The molecular formulas [Bi_38_O_45_(Boc-l-Met-O)_24_] and [Bi_38_O_45_(Boc-l-Phe-O)_24_] give molecular weights of M = 14620.9 g·mol^–1^ and M = 15004.4 g·mol^–1^ and the number of the valence electrons per formula unit of *n* = 2616 and *n* = 2856, respectively. Due to the complex nature of the hybrid organic–inorganic BiO-NCs, it is not possible to easily determine the density of the bulk material. We have therefore summarized the crystallographic densities of similar BiO-NCs in Table S6. The layer thickness was then calculated using different densities in the range between 2.5 and 3.5 g·cm^–3^; the values obtained change slightly within the error bar (see Figure S15). Therefore, the density value of (3.0 ± 0.3) g·cm^–3^ was used for further calculations. The XPS-estimated thicknesses are (4.3 ± 0.5) nm for **di2**_120w_ and (2.5 ± 0.5) nm for **di1**_120w_ (cf. Table 1). It should be noted that these values are larger than the values we obtained from the ellipsometry data, which are discussed in the following part. The discrepancy may partly originate from the assumption of a perfectly homogeneous closed film used for the XPS method. Furthermore, the samples were rinsed and transported in a normal air environment, so another reason for the larger XPS thickness values compared to those from SE is attributed to the presence of adventitious carbon,^37^ which additionally attenuates the Au XPS peaks and thus increases the calculated film thickness^47^ (cf. Table 1). The thickness of the adventitious carbon layer on the bare Au substrate identically treated to those used for the BiO cluster deposition is estimated to be (1.9–2.5) nm (cf. Table S7). Despite the difference in the absolute values, both methods (XPS and SE) confirm that the thickness of the BiO-NC **2** films is significantly larger compared to that of the BiO-NC **1** films.

**Table 1:** Film Thickness Estimation Using the XPS Method^a^

	peak name	peak KE (eV)	λ (nm)	peak area (a.u.)	*d* (nm)
**di1**_120w_	Au 4f	1399	2.89	1	2.5 ± 0.5
	Au 4p_3/2_	938	2.12	0.73	
**di2**_120w_	Au 4f	1399	2.86	1	4.3 ± 0.5
	Au 4p_3/2_	938	2.10	0.59	

a KE—kinetic energy, λ—inelastic mean free path of electron, *d*—overlayer thickness.

### 4.3 Cluster–Au Interface Characterization Using Spectroscopic Ellipsometry

In order to further analyze the adsorption of the clusters on Au depending on their composition and in order to determine the layer thickness independently, we used SE. Its optimal sensitivity is attainable when selecting an AOI near the Brewster angle. At this point, the Fresnel coefficient of reflection for incident light with polarization parallel to the plane of incidence *r*_p_ approaches zero, leading to increased sensitivity. Hence, all data presented are shown for an AOI = 65°. The measured ellipsometric parameters Ψ and Δ are directly related to the Fresnel reflection coefficients for parallel *r*_p_ and perpendicular *r*_s_ polarized light where Ψ can be assigned to the ratio between the amplitudes *r*_p_ and *r*_s_ (cf. eq 2), while Δ represents the phase shift between them
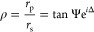
2

Since the Δ parameter is more sensitive to the sample thickness, the optical model (three-layer Arwin model^31^) was applied accordingly.

By comparing the ellipsometric spectra of thin films with those of the pristine substrate, the adsorption process leads to visible changes in the Ψ and Δ values even in the case when the adsorbent is only in the range of a few nm, as it is in the case of SAM on Au surfaces. Therefore, in such cases, it has become an established method to calculate the difference spectra as shown in eqs 3.1 and 3.2 for Ψ and Δ, respectively^48,49^
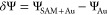
3.1
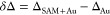
3.2

**Figure 5:**
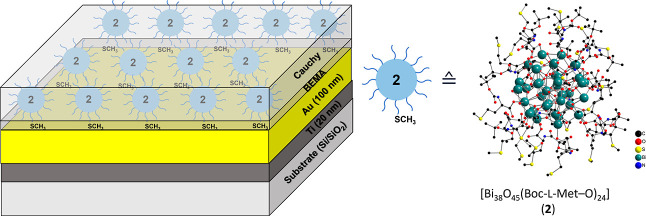
(*Left*) Layer-by-layer model for SAM, which was applied for the ellipsometric modeling for BiO-NCs **1** and **2**. The optical constants for the Au surface were extracted by applying a Kramers–Kronig consistent B-spline. The BEMA layer corresponds to the Au–S interaction, which arises at the interface. The Cauchy layer represents the transparent layer of self-assembled BiO-NCs **1** and **2**. (*Right*) Schematic molecular structure of BiO-NC **2**.

SAM can be associated with a self-assembled layer, which corresponds to the clusters assembling on the Au surfaces. To extract the film thickness, the three-layer Arwin model (schematically depicted in Figure 5) was used. At first, the Au-coated silicon substrate was modeled by a Kramers–Kronig consistent B-spline layer. The Au–S interaction, which arises at the interface, was modeled using an Bruggeman effective medium approximation (BEMA) with an approximate thickness of *t*_BEMA_ = 0.2 nm, which was taken from the literature.^6^ The volume ratio of 1:1 [with material 1 = Au and material 2 = BiO-NCs (represented by a Cauchy layer)] was kept fixed according to this reference. The optical constants of the self-assembled layers of the BiO-NC **1** or **2** were parametrized by a Cauchy layer, with the literature values A, B, and C parameters^6^ (see details in Table S8). To obtain the changes induced by the deposition of BiO-NCs, the samples were rinsed by spectroscopic-grade ethanol after the dipping procedure (cf. Table S1) to ensure the removal of all weakly bonded residuals.

**Figure 6:**
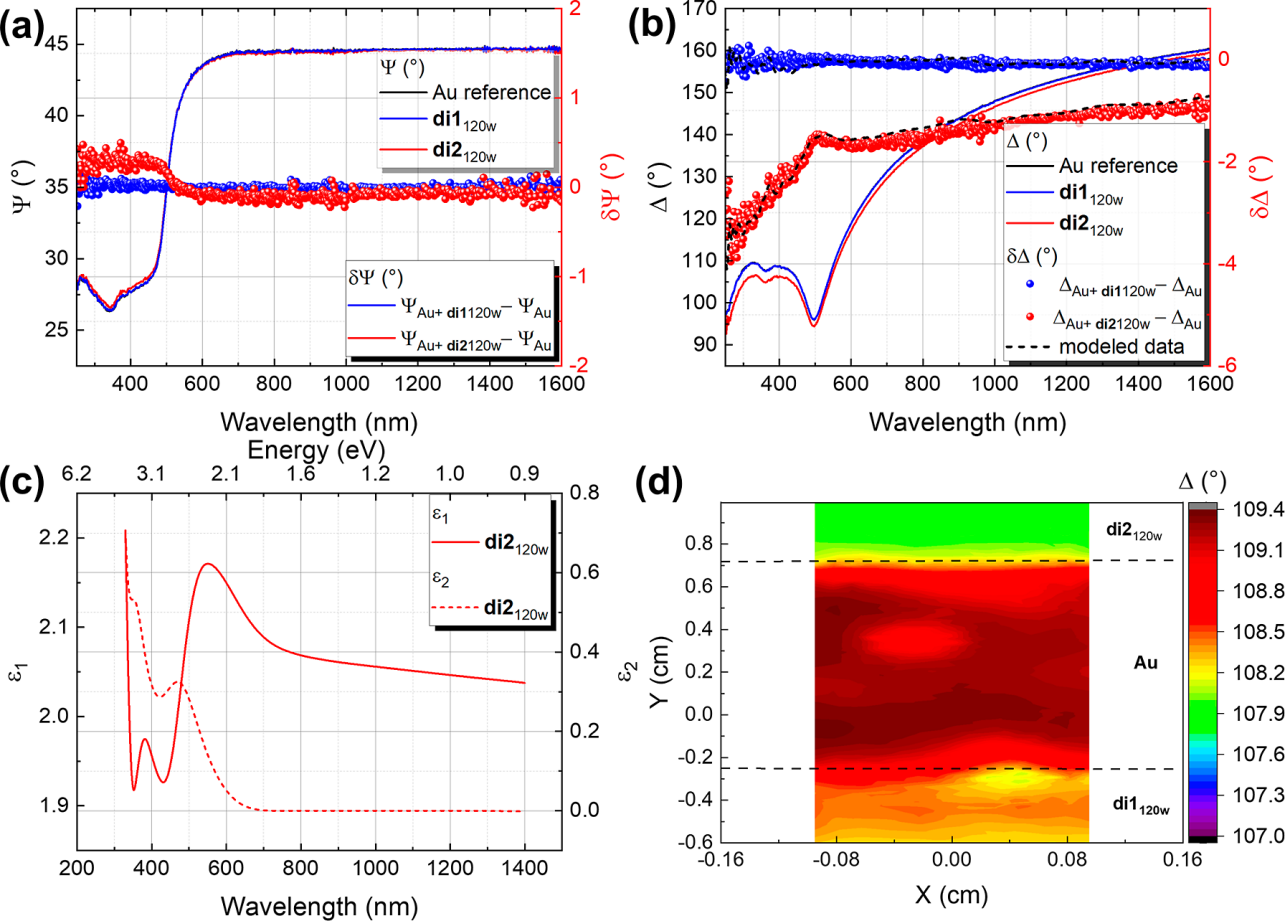
(a) Ψ and difference δΨ spectra for **di1**_120w_ (blue curve and blue dotted curve) compared to the spectra obtained for **di2**_120w_ after 2 h of dipping time in solution of the clusters and absolute ethanol washing. (b) Δ and difference δΔ spectra for **di1**_120w_ and **di2**_120w_. (c) Dielectric function for the BiO-NC **2** under consideration of the Au–S interaction determined by SE modeling using a Cauchy layer for the nonabsorbing range of 600–1400 nm. The extension to the absorbing range <600 nm was performed using a B-spline function with a total MSE = 2.64. (d) Mapping across three samples underneath each other, namely, **di2**_120w_, Au surfaces, and **di1**_120w_ BiO-NCs for 2 h dipping time (from top to bottom), respectively. Measurement taken at an AOI = 65°, where the alignment was performed at each position.

A map was taken across three samples, namely, **di2**_120w_, uncovered Au, and **di1**_120w_ (from top to bottom, cf. Figure 6d), to observe the uniformity of adsorbed cluster molecules and to compare the adsorption strength depending on the ligand choice.

The results for the BiO-NC **di1**_120w_ and **di2**_120w_ obtained for the δΨ and δΔ as well as the pure data for Ψ and Δ are displayed exemplarily in Figure 6a,b, respectively (extracted from the map at the marked positions). In the δΨ difference spectra taken for **di2**_120w_, a transition at ∼500 nm is observed with positive values in the spectral range from 250–500 nm and slightly negative or zero values were acquired at larger wavelengths. A similar behavior was previously observed when a strong interaction between thiol-containing molecules and Au surfaces was present.^6,50^ For those molecules, the above-mentioned optical transition was assigned to metal-induced electronic interface states in the highest occupied molecular orbital–lowest unoccupied molecular orbital gap of the molecules by the formation of the S–Au bond.

The occurrence of this feature was related with two hybrid virtual states of the S–Au interface. The higher and lower energy states are the σ* orbital and an antibonding state between sulfur and the Au surfaces, respectively.^6,51^ In the case of **di2**_120w_, the observation of the above-mentioned transition thus proves the existence of a Au–S interface interaction. Since no such transition was observed for **di1**_120w_, we can also conclude that no strong chemical interaction was formed between the Boc-l-phenylalanine ligands and Au surfaces and that the BiO-NC **1** is more likely to be bonded via van der Waals interactions. Since BiO-NC **1** does not exhibit a sulfur-containing functional group, the result is in line with our expectation as well as with the literature.^48^ For BiO-NC **2**, the layer thickness could be extracted using the three-layer Arwin model,^31^ wherefore the parameters for the Cauchy layer were chosen according to Table S7.^6^ The total thickness of the BiO-NC **2** layer was determined to be *t*_total_ = (1.86 ± 0.02) nm [mean square error (MSE) = 3.86] (cf. Figure 6b). Thus, the calculated total layer thickness of BiO-NC **2** agrees well with the calculated layer spacing in the solid state of 1.98 nm (cf. PXRD Figure S5) as well as with the slightly larger hydrodynamic diameter of 2.5 nm measured in solution (cf. DLS Figure S6). As can be determined from the ellipsometry data modeling for **di1**_120w_ (cf. Figure 6b), the three-layer Arwin model cannot be applied in this case. The absence of a strong chemical interaction between the Au surface and the Boc-l-phenylalanine ligands does not lead to an effective thickness for the BEMA layer. In this respect, the model reaches its limits because it yields an unrealistic total thickness of the **di1**_120w_ of *t*_total_ = (0.05 ± 0.01) nm (MSE = 2.68), where the BEMA layer was set to *t*_BEMA_ = 0 nm. The small difference in the spectra taken before and after the deposition can occur due to changes in the optical response, related to the dipping procedure or the attachment of organic residuals to the Au surfaces. Lower Δ values in comparison to those of the pristine Au surfaces were observed for the whole sample **di2**_120w_ (cf. map in Figure 6d). This indicates an increased and homogeneous layer thickness. Accordingly, the attachment seems to take place uniformly only for the sample containing Boc-l-methionine ligands with its sulfur-comprising functional group. Nevertheless, it is worth mentioning that BiO-NC **1** remained at some spots of the surfaces after rinsing, leading to slightly decreased Δ values (compared to Δ_Au_). As the Cauchy layer does not take the absorption of the cluster into account, the model was extended with an additional B-spline function in the range below 600 nm. This allowed the dielectric function for BiO-NC **2**, under consideration of the Au–S interaction, to be determined as displayed in Figure 6c. The imaginary part (ε_2_) displays the absorption behavior of BiO-NC **2** (+Au–S interface), where a feature at 471 nm (≙ 2.63 eV) can be observed. Since the parametrized Cauchy layer is coupled to the underlying BEMA layer, it can be expected that this feature is related to the Au–S interface. According to the literature,^52^ a similar interface layer absorption was related to nanoscale morphological modifications arising from the formation of Au–S bonding. It was previously suggested for alkanethiols by Prato et al.^53^ that the behavior originates from a reduction of the mean free path of Drude electrons or a red shift of the plasmonic mode in the near-surface region. The dielectric function for BiO-NC **1** could not be extracted, since the model considers a full coverage, which is not the case for **di1**_120w_ (cf. Figure 6d). Hence, the calculation revealed in this case an unrealistic thickness (*t*_B-spline_ = *t*_total_) value below 1 nm (MSE = 3.21), while the BEMA thickness *t*_BEMA_ was kept constant at 0 nm.

## 5 Conclusions

Despite the increasing interest in CISS and related technological applications, the main research focus has been on chiral molecules and biomolecules and their interfaces. In this study, we discuss the feasibility of chiral nanoclusters for the self-assembling on Au surfaces. The successful synthesis of the chiral atomically precise BiO-NC [Bi_38_O_45_(Boc-l-Met-O)_24_] (**2**) was demonstrated using several analytical techniques such as DLS, ESI-MS, XRD, and IR spectroscopy. The chiral nature was proven by CD spectroscopy. The adsorption capability of the thioether anchor group containing BiO-NC **2** on the Au surface was investigated in comparison to the previously prepared BiO-NC [Bi_38_O_45_(Boc-l-Phe-O)_24_(dmso)_9_] (**1**). By SEM, SE, and AFM, we showed that the homogeneity and uniformity of the films of BiO-NCs on Au strongly depend on the choice of the amino acid-derived ligands at the cluster shell. For BiO-NC **2**, a specific spectral feature was observed by SE, which demonstrates a sufficiently strong chemical Au–S interaction between the gold surfaces and the BiO-NC, which enables the formation of an SAM. This spectral feature was not present for films prepared from BiO-NC **1** on Au, which is consistent with a rather weak van der Waals interaction between BiO-NC **1**(48) and the Au surface. The effective thickness of the layer BiO-NC **2** calculated by SE as well as XPS under consideration of adventitious carbon corresponds to an SAM, which was estimated to be ∼2 nm.^13^

The successful self-assembling of the BiO-NCs and their strong interaction with the Au substrate make them promising candidates for CISS-related applications, for example, in the field of opto-spintronics, where chiral atomically precise BiO-NCs could be used for light-induced electron spin polarization, for example in devices sich as hybrid magnetic tunnel junctions (based on the principle proposed in ref (54)). Furthermore, we believe that this versatile and simple approach for anchoring chiral BiO-NCs on metallic surfaces could be extended to a range of other chiral nanoclusters and nanoparticles.

## Data Availability

All data generated or analyzed during this study are included in this published article and its Supporting Information. The raw data is available upon request.

## Supporting Information Available

Sample assignment with the corresponding sample preparation method; results of the full BiO-NC characterization performed by CD, UV–vis spectroscopy, ESI-mass spectrometry, PXRD, DLS, NMR, and IR spectroscopy; analysis of the unwashed and washed films via IR spectroscopy, XRD, XPS as well as by AFM and SEM investigations; overview of the crystallographic density of different BiO-NCs as well as the full XPS layer thickness calculation; and detailed description for the parameters used in the three-layer Arwin model (PDF)
