# Supplementary material for: Anchoring Atomically Precise Chiral Bismuth Oxido Nanoclusters on Gold: The Role of Amino Acid Linkers
Source: Langmuir. 2024 Jul 12;40(31):16320–9. doi: 10.1021/acs.langmuir.4c01445 (PMC11308521; doi:10.1021/acs.langmuir.4c01445)
Supplement: Supplementary file 1 — la4c01445_si_001.pdf [file la4c01445_si_001.pdf]

Supplementary Information

Anchoring atomically precise chiral bismuth oxido nanoclusters on gold: The role of amino acid linkers

Annika Morgenstern[1,⊥], Rico Thomas[2,⊥], Oleksandr Selyshchev[1,4], Marcus Weber[2,4], Christoph Tegenkamp[3], Dietrich R.T. Zahn[1,4], Michael Mehring[2,4*], Georgeta Salvan[1,4*]

[1]Faculty of Natural Science, Institute of Physics, Semiconductor Physics, Chemnitz University of Technology, 09107 Chemnitz, Germany

[2]Faculty of Natural Science, Institute of Chemistry, Coordination Chemistry, Chemnitz University of Technology, 09107 Chemnitz, Germany

[3]Faculty of Natural Science, Institute of Physics, Analysis of Solid Surfaces, Chemnitz University of Technology, 09107 Chemnitz, Germany

[4]Center of Materials, Architectures and Integration of Nanomembranes, Chemnitz University of Technology, 09126 Chemnitz, Germany

E-Mail: salvan@physik.tu-chemnitz.de and michael.mehring@chemie.tu-chemnitz.de

Supplementary information

1. Materials and Methods.....2
2. BiO-NC characterization.....2
2.1. CD spectroscopy.....2
2.2. UV-vis results BiO-NC 1 and BiO-NC 2.....2
2.3. ESI-MS results.....3
2.4. PXRD and PSD (DLS) results.....5
2.5. NMR results.....6
2.6. IR results.....7
3. Film characterization.....8
3.1. IR results.....8
3.2. XRD results.....9
3.3. XPS results.....9
3.4 AFM results.....11
3.5. SEM results.....11
3.6. Overview crystallographic density of different BiO-NCs.....12
3.7. XPS layer thickness calculation.....12
3.8. SE- fitting parameters for Arwin model.....13
References.....13

1. Materials and Methods

Table S 1. Sample assignment for BiO-NCs and resulting BiO-NC films together with the corresponding sample preparation method.

Sample	Nanocluster description	Sample preparation method
**1**	$[Bi_{38}O_{45}(\text{Boc-L-Phe–O})_{24}(\text{dmso})_9]$[1]	Chemical synthesis (microcrystalline powder)
**2**	$[Bi_{38}O_{45}(\text{Boc-L-Met–O})_{24}]$	Chemical synthesis (microcrystalline powder)
**di1**$_{120}$	$[Bi_{38}O_{45}(\text{Boc-L-Phe–O})_{24}(\text{dmso})_9]$	Substrate dipped for 2 h in ethanol solution of BiO-NC **1** and used without further treatment
**di1**$_{120w}$		Substrate dipped for 2 h in ethanol solution of BiO-NC **1** and rinsed afterwards three times with spectroscopic grade ethanol
**di2**$_{120}$	$[Bi_{38}O_{45}(\text{Boc-L-Met–O})_{24}]$	Substrate dipped for 2 h in ethanol solution of BiO-NC **2** and used without further treatment
**di2**$_{120w}$		Substrate dipped for 2 h in ethanol solution of BiO-NC **2** and rinsed afterwards three times with spectroscopic grade ethanol

2. BiO-NC characterization

2.1. CD spectroscopy

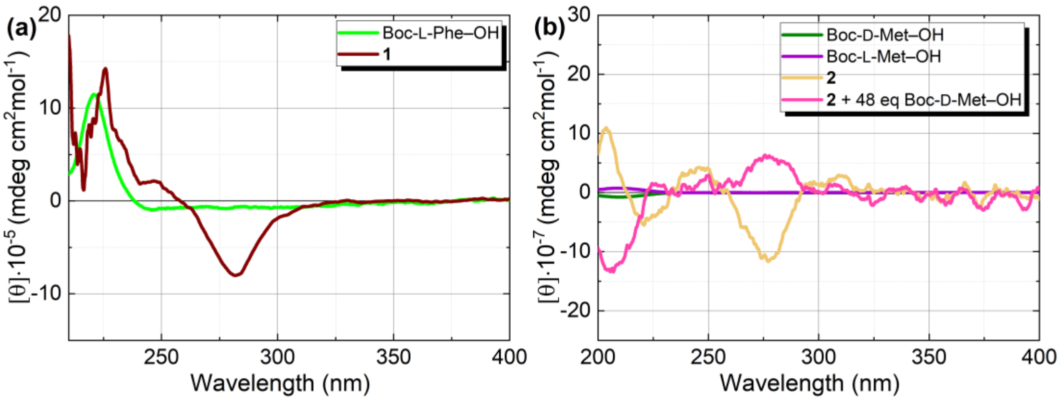

Figure S 1. (a) CD spectra of Boc-L-Phenylalanine (c = $1{\cdot}10^{-3}$ M) and BiO-NC **1** (c = $1{\cdot}10^{-5}$ M) in Acetonitrile;(b) CD spectra of Boc-D-methionine (c = $1{\cdot}10^{-3}$ M), Boc-L-methionine (c = $1{\cdot}10^{-3}$ M), BiO-NC **2** (c = $1{\cdot}10^{-5}$ M) and BiO-NC **2** +48 eq Boc-D-methionine (c = $1{\cdot}10^{-5}$ M)in acetonitrile.

The transfer of the chiral information from the Boc-L-Phenylalanine to the BiO-NC **1** was proven using CD spectroscopy. Spectra of cluster **1** exhibit a Cotton effect signal in the range of 250 nm – 325 nm with a maximum at 282 nm similar to those of the previously published cluster $[Bi_{38}O_{45}(\text{Boc-L-Phe–O})_{22}(OH)_2]$[2]. The spectra of the respective Boc-D-Phe–OH is displayed as well in ref.[2] showing the expected signal in the opposite direction compared to Boc-L-Phe–OH. Therefore, the transfer of the chiral information from Boc-L-Phe–OH to cluster **1** was demonstrated.

In order to prove the possibility of synthesizing the opposite enantiomer we performed an in-situ ligand exchange reaction. Starting from BiO-NC **2** $[Bi_{38}O_{45}(\text{Boc-L-Met–O})_{24}]$ the Boc-D-Met–OH amino acid was added in excess (48 eq amino acid) and the resulting spectrum was measured after 2 min of stirring at room temperature. In addition to the signal for the D-enantiomer of the free Boc-D-Met–OH amino acid at 207 nm the Cotton effect signal for the cluster at 277 nm changed to the opposite direction. This result is in line with our previous results[2] and confirm the ligand exchange in solution.

2.2. UV-vis results BiO-NC 1 and BiO-NC 2

The absorption of the BiO-NC **1** and **2** powder was measured by UV–vis spectroscopy. The band gap was calculated using Tauc plot with an exponent of $n = 1/2$[3], assuming a direct allowed band transition.

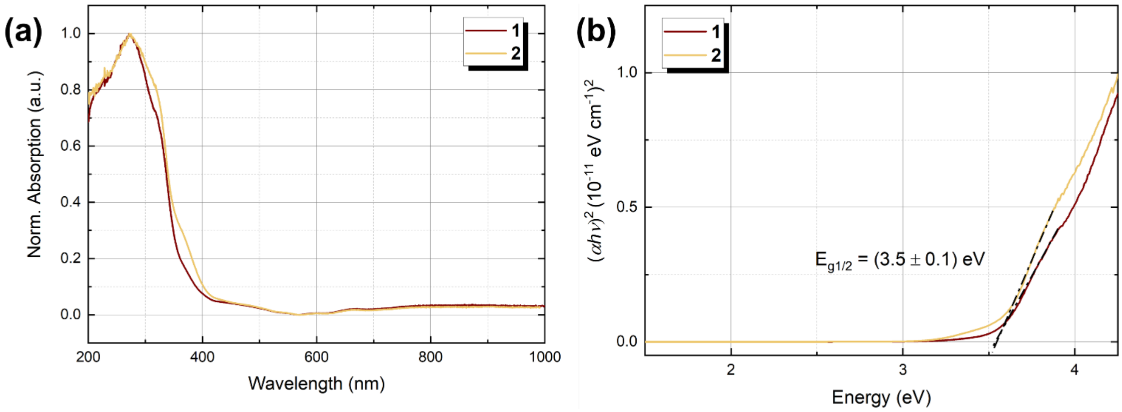

Figure S 2. (a) UV–vis absorption for BiO-NC 1 and 2 powder and (b) Tauc plot with extracted energy band gap for BiO-NC **1** and **2**, respectively. The exponent n = 1/2 was chosen for the calculation, as a direct band gap with allowed transitions is assumed for the cluster structure.

2.3. ESI-MS results

The chemical composition of BiO-NC **2** was proven by ESI mass spectrometry. Electrospraying of BiO-NC **2** from MeOH results in the generation of doubly to quadruply positively charged bismuth oxido cluster cations without additional solvates. All assigned BiO-NC **2** cations as well es their m/z signals are summarized in Table S2. Survey spectra as well selected isotopic patterns of doubly positively charged cationic species of BiO-NC **2** are exemplarily shown in Figure S3. and Figure S4., the resulting cations gain their charge during the electrospray ionization by the loss of appropriate number of Boc-L-Met–O$^-$ counterions; combined in several cases with additional sodium carboxylate units. Selected isotopic patterns of most relevant species with high intensity were assigned exemplarily.

Table S 2. Selection of positively charged bismuth oxido nanocluster cations detected in the survey mass spectrum of BiO-NC 2 electrosprayed from MeOH. Assignment was carried out using the most abundant *m/z* signals.

Cation	Caption	*m/z*	
		exp.	calcd.
$[Bi_{38}O_{45}(\text{Boc-L-Met–O})_{23}Na]^{2+}$	I	7197.6169	7197.6104
$[Bi_{38}O_{45}(\text{Boc-L-Met–O})_{22}(\text{L-Met–O})Na]^{2+}$	II	7147.5673	7147.5841
$[Bi_{38}O_{45}(\text{Boc-L-Met–O})_{22}(OH)Na]^{2+}$	III	7081.5711	7081.5673
$[Bi_{38}O_{45}(\text{Boc-L-Met–O})_{22}]^{2+}$	IV	7061.5785	7061.5674
$[Bi_{38}O_{45}(\text{Boc-L-Met–O})_{21}(\text{L-Met–O})]^{2+}$	V	7011.5287	7011.5411
$[Bi_{38}O_{45}(\text{Boc-L-Met–O})_{20}(\text{L-Met–O})_2]^{2+}$	VI	6961.5246	6961.5148
$[Bi_{38}O_{46}(\text{Boc-L-Met–O})_{21}Na]^{2+}$	VII	6957.0211	6957.0119
$[Bi_{38}O_{45}(\text{Boc-L-Met–O})_{21}(OH)]^{2+}$	VIII	6946.0298	6946.0209
$[Bi_{38}O_{46}(\text{Boc-L-Met–O})_{20}(\text{L-Met–O})Na]^{2+}$	IX	6906.9884	6906.9856
$[Bi_{38}O_{45}(\text{Boc-L-Met–O})_{20}(\text{L-Met–O})(OH)]^{2+}$	X	6895.9780	6895.9946
$[Bi_{38}O_{46}(\text{Boc-L-Met–O})_{20}]^{2+}$	XI	6821.4829	6821.4691
$[Bi_{38}O_{46}(\text{Boc-L-Met–O})_{19}(\text{L-Met–O})]^{2+}$	XII	6771.4434	6771.4428
$[Bi_{38}O_{45}(\text{Boc-L-Met–O})_{22}Na]^{3+}$	XIII	4715.3775	4715.3747
$[Bi_{38}O_{45}(\text{Boc-L-Met–O})_{21}(\text{Boc-L-Met–O})Na]^{3+}$	XIV	4682.0028	4682.0238
$[Bi_{38}O_{45}(\text{Boc-L-Met–O})_{22}Na_2]^{4+}$	XV	3542.2655	3542.2783
$[Bi_{38}O_{45}(\text{Boc-L-Met–O})_{21}(\text{Boc-L-Met–O})Na_2]^{4+}$	XVI	3517.2448	3517.2652

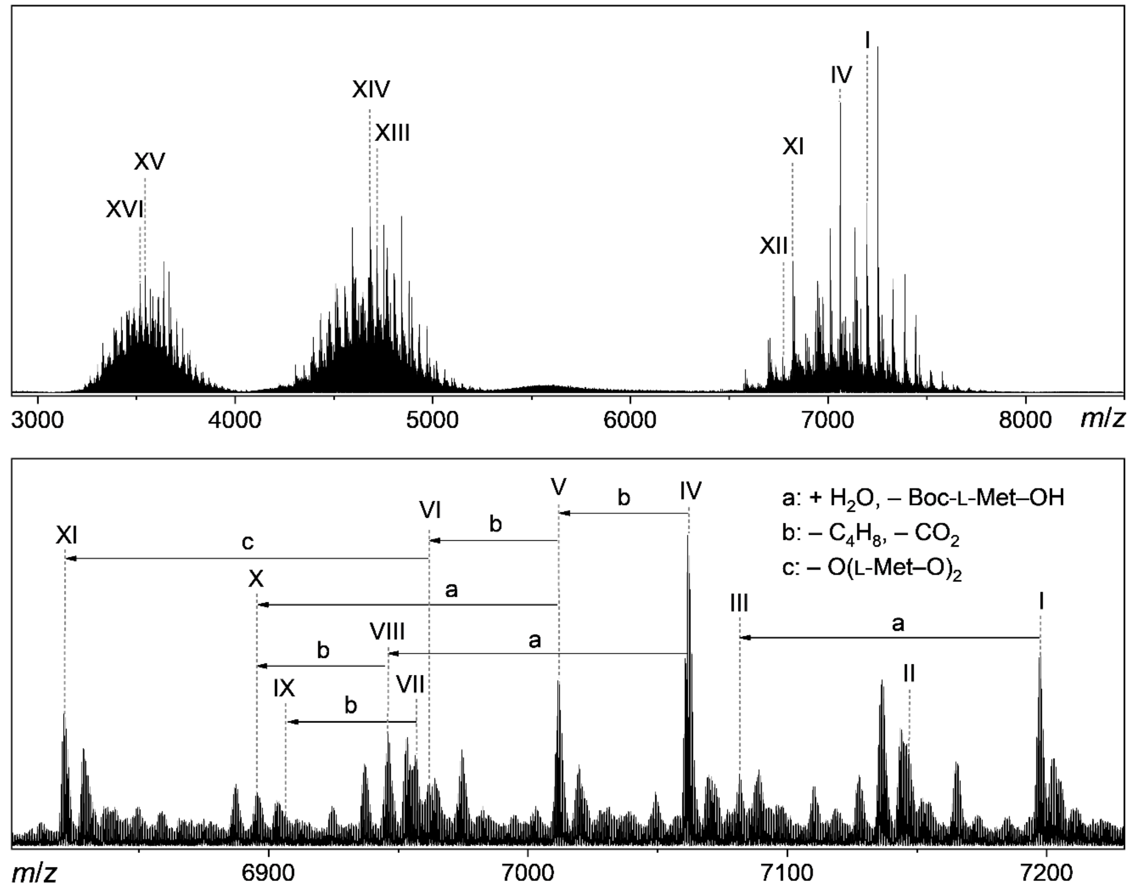

Figure S 3. *Top*: Survey mass spectrum of [$Bi_{38}O_{45}$(Boc-L-Met–O)$_{24}$] (**2**) electrosprayed from MeOH showing typical doubly (I–XII), triply (XIII, XIV), and quadruply (XV, XVI) positively charged bismuth oxido nanocluster cations (see Table S2.) *Bottom*: Cutout of the ESI mass spectrum (m/z = 6800 – 7225) of the BiO-NC **2** showing signals assigned to doubly positively charged species such as [$Bi_{38}O_{45}$(Boc-L-Met–O)$_{22}$]$^{2+}$ (IV, m/z = 7061.5785), and partially Boc-deprotected fragments like [$Bi_{38}O_{45}$(Boc-L-Met–O)$_{21}$(L-Met–O)]$^{2+}$ (V, m/z = 7011.5287).

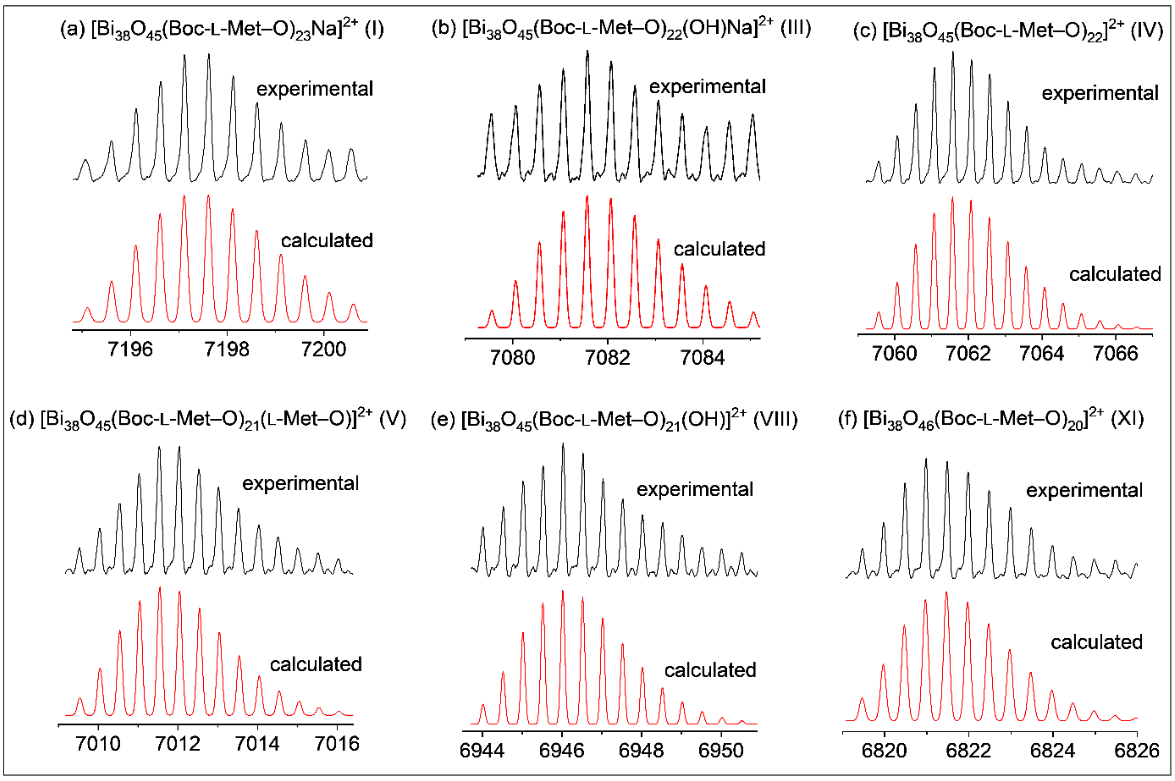

Figure S 4. Isotopic patterns (exp. and calcd.) of selected doubly positive charged bismuth oxido nanocluster cations detected in the gas phase generated from compound **2** after electrospraying from MeOH. (a) $[Bi_{38}O_{45}(Boc\text{-}L\text{-}Met–O)_{23}Na]^{2+}$ (I), (b) $[Bi_{38}O_{45}(Boc\text{-}L\text{-}Met–O)_{22}(OH)Na]^{2+}$ (III), (c) $[Bi_{38}O_{45}(Boc\text{-}L\text{-}Met–O)_{22}]^{2+}$ (IV), (d) $[Bi_{38}O_{45}(Boc\text{-}L\text{-}Met–O)_{21}(L\text{-}Met–O)]^{2+}$ (V), (e) $[Bi_{38}O_{45}(Boc\text{-}L\text{-}Met–O)_{21}(OH)]^{2+}$ (VIII), (f) $[Bi_{38}O_{46}(Boc\text{-}L\text{-}Met–O)_{20}]^{2+}$ (XI).

After electrospraying of BiO-NC $[Bi_{38}O_{45}(Boc\text{-}L\text{-}Met–O)_{24}]$ (**2**) from MeOH, doubly positive charged cations such as $[Bi_{38}O_{45}(Boc\text{-}L\text{-}Met–O)_{23}Na]^{2+}$ (I) and $[Bi_{38}O_{45}(Boc\text{-}L\text{-}Met–O)_{22}]^{2+}$ (IV) were successfully assigned as most intense species. However, even under the soft ionization conditions applying electrospraying, ongoing elimination of Boc-protecting groups (as isobutene and carbon dioxide) takes place commonly known from preliminary reports[2, 4]. After the Boc-group has been eliminated twice from precursor IV to give $[Bi_{38}O_{45}(Boc\text{-}L\text{-}Met–O)_{20}(L\text{-}Met–O)_2]^{2+}$ (VI), the typical loss of carboxylic acid anhydride $O(L\text{-}Met–O)_2$, a well-known fragmentation reaction for carboxylate-modified BiO-NCs like $[Bi_{38}O_{45}(OMc)_{24}]$ (OMc = $^-O_2C_4H_5$),[5] results in the formation of the fragment successfully assigned as $[Bi_{38}O_{46}(Boc\text{-}L\text{-}Met–O)_{20}]^{2+}$ (XI). Noteworthy, partially hydrolyzed species such as $[Bi_{38}O_{45}(Boc\text{-}L\text{-}Met–O)_{21+x}(OH)Na_x]^{2+}$ (x = 1, 0; III, VIII) were detected, too; the hydrolysis might be caused either by the previous washing procedure of BiO-NC **2** using deionised water or during the ESI-MS measurement Noteworthy, partial hydrolysis of BiO-NCs coordinated by Boc-protected amino acids and the detection in the gas phase was demonstrated in a former work[2]. Please note, that solely fragments with maximum of one $^-$OH group and low abundance were identified, indicating a low impact of hydrolysis for BiO-NC **2**.

2.4. PXRD and PSD (DLS) results

The recorded PXRD patterns of the as-prepared bismuth oxido clusters are shown in the following Figure S5. All samples show the typical reflection patterns, whereby the intact $[Bi_{38}O_{45}]$ cluster core is confirmed via detection of highly intense reflection in the range of (4 – 6)° 2θ.[6] The so-called "main diffraction peaks" of BiO-NC **2** correspond to the interlayer distance between closed packed molecule arrangement, which is shifted to lower 2θ values compared to precursor compound **A** indicating a larger unit cell of BiO-NC **2**. Similar results were observed for BiO-NC **1**, which is in line with the expectations according to the coordination of larger ligands.[1] The main diffraction values and therefore interlayer distances for all BiO-NCs were determined for **A** with (2θ = 5.23 °; d = 1.69 nm), **1** with (2θ = 4.47 °; d = 1.98 nm), and **2** with (2θ = 4.46 °; d = 1.98 nm). The calculated interlayer distance for the BiO-NC is in good agreement with hydrodynamic diameter "d_h" based on dynamic light scattering (DLS) experiments of the above-mentioned compounds in solution and calculated from the fitted particle size distribution (PSD) curve. The hydrodynamic diameter of BiO-NC **2** (Figure S6.) in ethanol and dmso was determined as $d_h \approx 2.5$ nm, which is in the same range as observed for BiO-NC **1** showing values d_h = (2.2 – 2.9) nm.

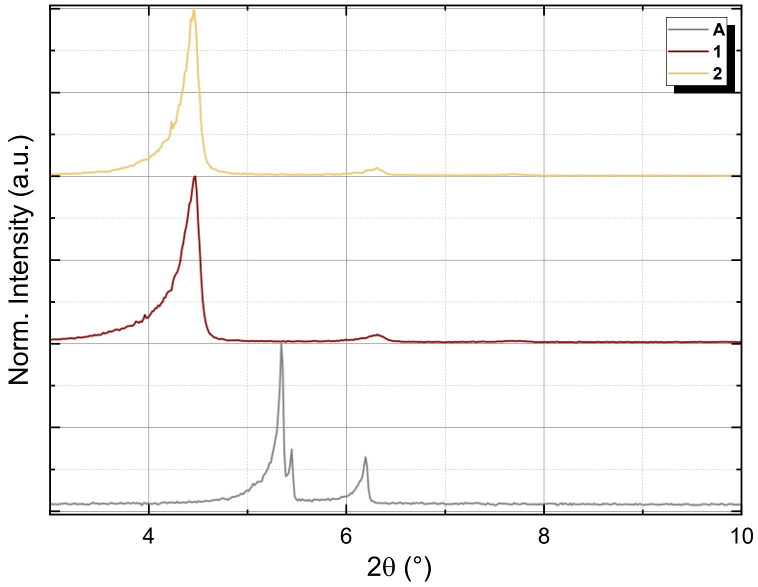

Figure S 5. PXRD pattern of bulk samples of cluster **A** (grey), BiO-NC **1** (red) and BiO-NC **2** (orange).

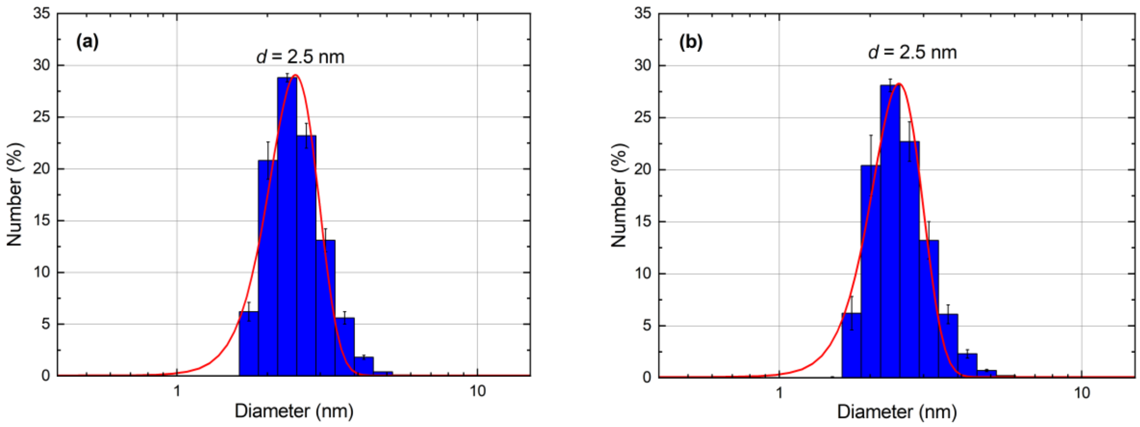

Figure S 6. Exemplary particle size distribution (PSD) determined from DLS of BiO-NC **2** in ethanol (a) and dmso (b).

2.5. NMR results

NMR spectra (Figure S7) confirmed the ligand exchange from nitrate to the Boc-protected-L-methionine ligand. ^{1}H NMR (Figure S7. (a)) and ^{13}C NMR spectra (Figure S7 (b)) show all expected signals for Boc-L-Met–O^{-}. A signal for the coordinating DMSO can still be detected as a minor impurity.

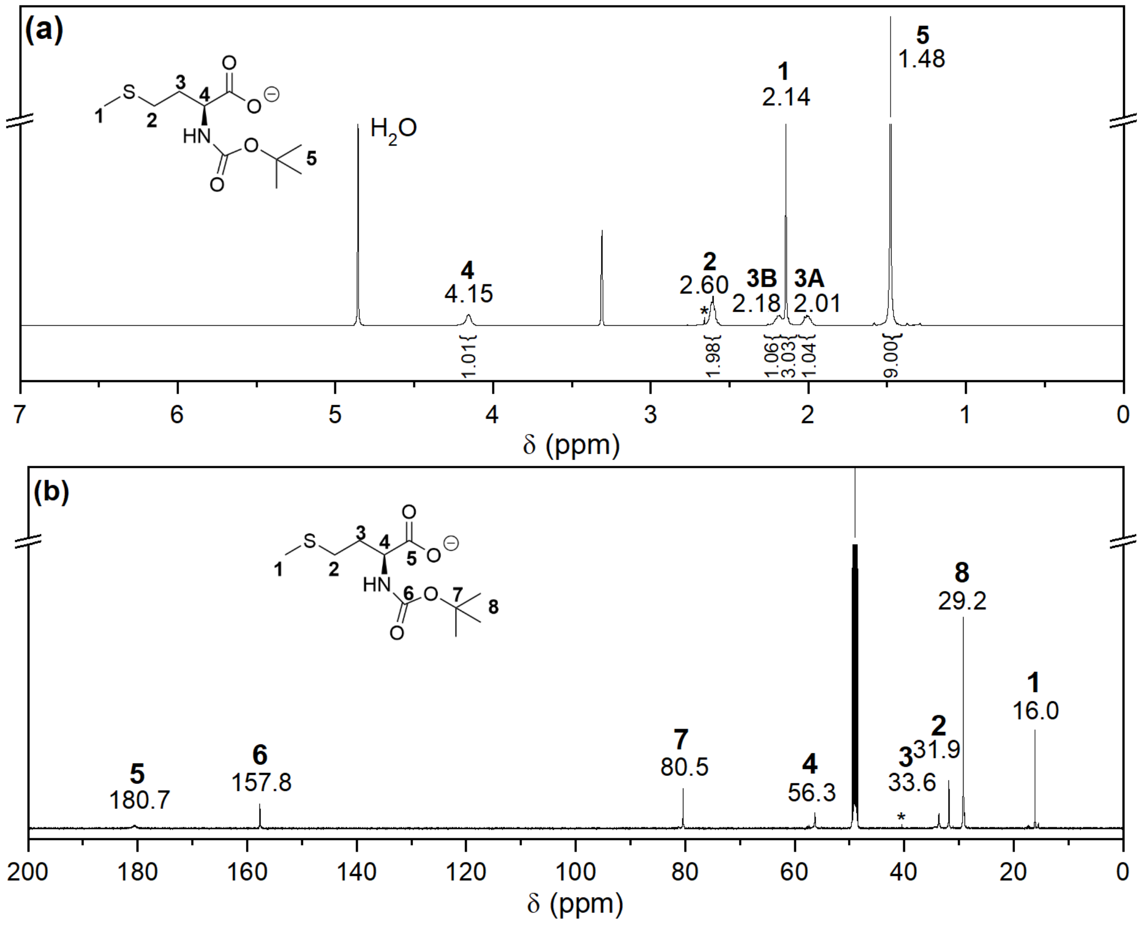

Figure S 7. (a) ^{1}H NMR spectra (500.3 MHz, 298 K) of 2 in MeOD-d$_4$. (b) ^{13}C NMR spectra (125.8 MHz, 298 K) of **2** in MeOD-d$_4$. Signals marked with * are assigned residuals of DMSO.

2.6. IR results

The ATR-IR measurements (Figure S8**Figure S 8**) of **C** and BiO-NC **2** result in similar spectra and provide all expected vibrations for the Boc-protected-L-methionine with minor shifts in the peak position and broader peaks for **2** due to the different coordination environment in cluster and sodium salt. The major differences in the spectra of BiO-NC **2** becomes obvious for the OH and NH vibration above 3000 cm^{-1}, which turned out to be drastically broader, and the additional broad Bi–O vibration at 540 cm^{-1}. More intensive vibrations in the fingerprint area are present but cannot be assigned due to their similarity in position (cf. Table S3.) nevertheless, they confirm the presence of the Boc-L-methionine ligand. Comparison of BiO-NC **2** with the starting material **A** (cf. Figure S9.) indicates the complete substitution of NO_3^- due to the absence of nitrate; monodentate coordinating nitrate ($\nu_{as}(NO_2)$: 1432 cm^{-1}, $\nu_s(NO_2)$: 1000 cm^{-1}); bidentate coordinating nitrate ($\nu_{as}(NO_2)$: 1382 cm^{-1}, $\nu_s(NO_2)$: 1266 cm^{-1}); and non-coordinating nitrate (ν(N=O): 1741 cm^{-1}, 1640 cm^{-1}) are observed for **A**.[7-8]

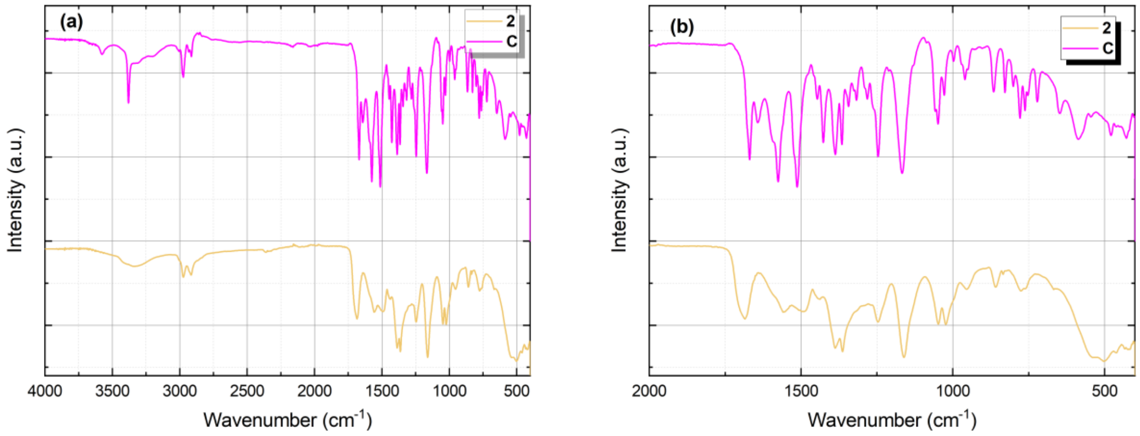

Figure S 8. Comparison of the ATR-FTIR spectra left (a, full spectra) and right (b, cut-out) of Boc-L-methionine sodium salt **C** (purple) and BiO-NC **2** (orange).

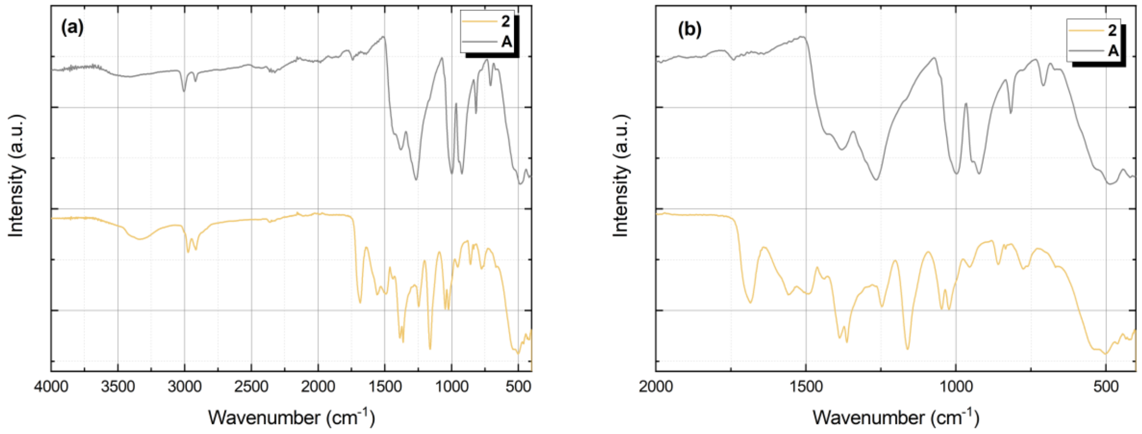

Figure S 9. Comparison of the ATR-FTIR spectra left (a, full spectra) and right (b, cut-out) of bismuth oxido nanoclusters **A** (grey) and **2** (orange).

3. Film characterization

3.1. IR results

Table S 3. ATR-FTIR strong vibrational modes of Boc-L-methionine sodium salt (C), BiO-NC 2 and dip-coated film di2$_{120}$.

vibrational mode	Wavenumber (cm^{-1})		
	**C**	**2**	**di2**$_{120}$
$\nu_{C\text{–}H}$	2974, 2914	2974, 2915	2973, 2919
$\nu_{C=O\ Amid}$	1669	1684	1701
ν_{COO^-} ; δ_{CH3}	1575, 1512, 1387, 1365	1559, 1491, 1388, 1363	1558, 1489, 1390, 1365
$\nu_{C\text{–}O}$; $\nu_{C\text{–}N}$	1247, 1167, 1049, 1027	1247, 1161, 1048, 1023	1247, 1168, 1049, 1024

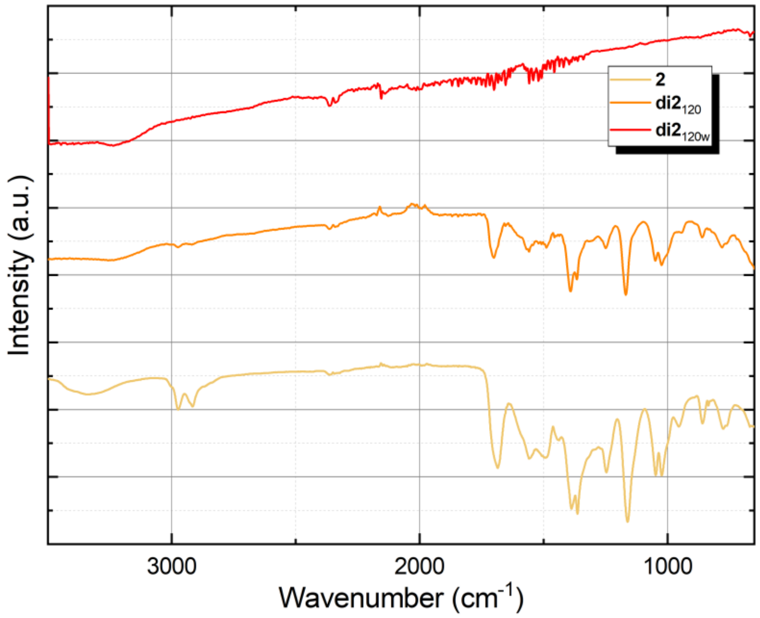

Figure S 10. IR spectra of BiO-NC film **di2**$_{120w}$ (*top*), of BiO-NC film **di2**$_{120}$ (*middle*) and bulk material of BiO-NC **2** as powdered sample (*bottom*).

3.2. XRD results

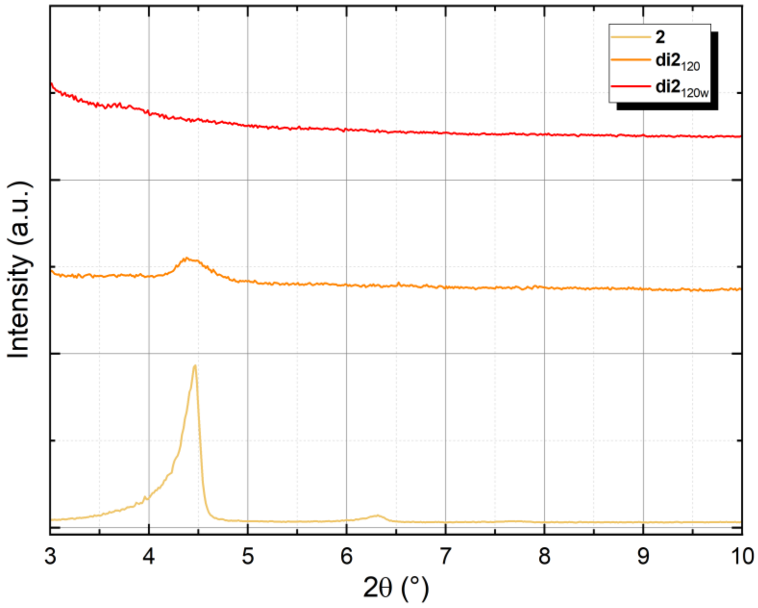

Figure S 11. XRD pattern of BiO-NC **2** powder (*bottom*), film **di2**$_{120}$ (*middle*) and film after rinsing with ethanol **di2**$_{120w}$ (*top*).

3.3. XPS results

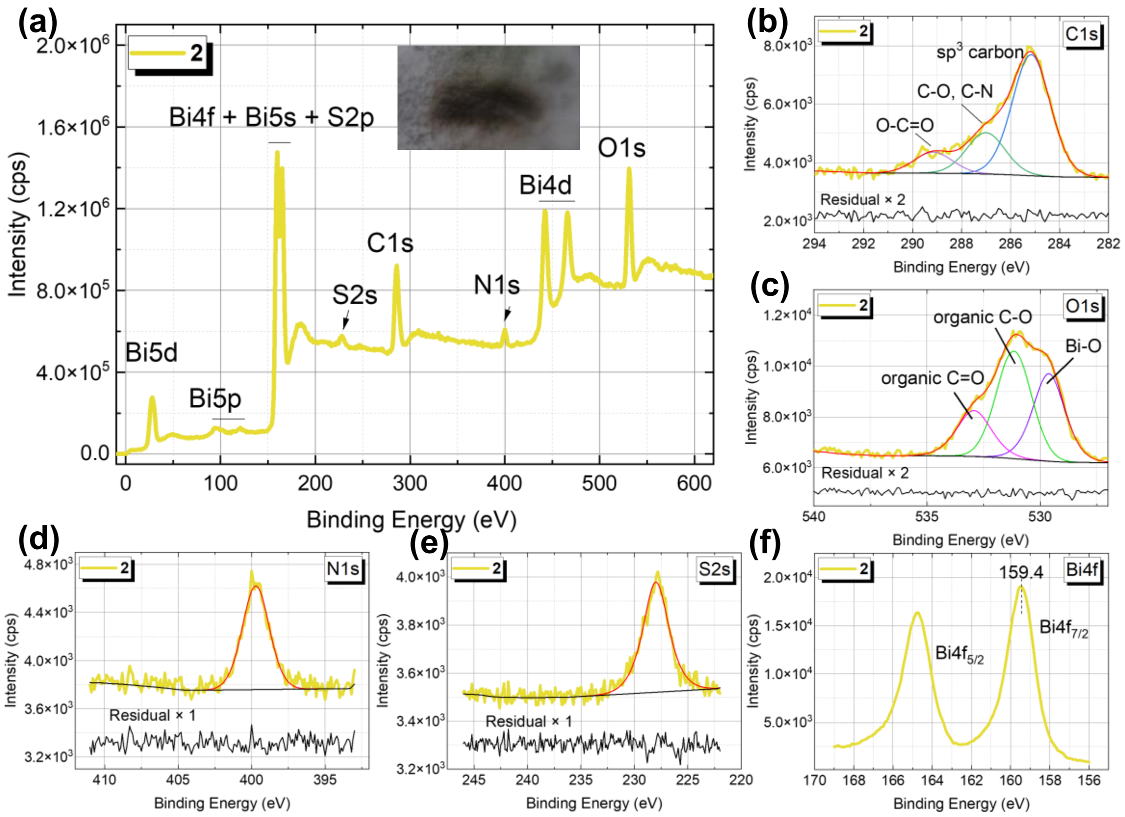

Figure S 12. (a) XPS of BiO-NC **2** powder sample. A fragment of XPS survey spectrum and the X-ray and charge compensation beams exposed sample area (insert). The color change indicates at least partial sample degradation. High resolution spectra for (b) C1s, (c) O1s, (d) N1s, (e) S2s and (f) Bi4f.

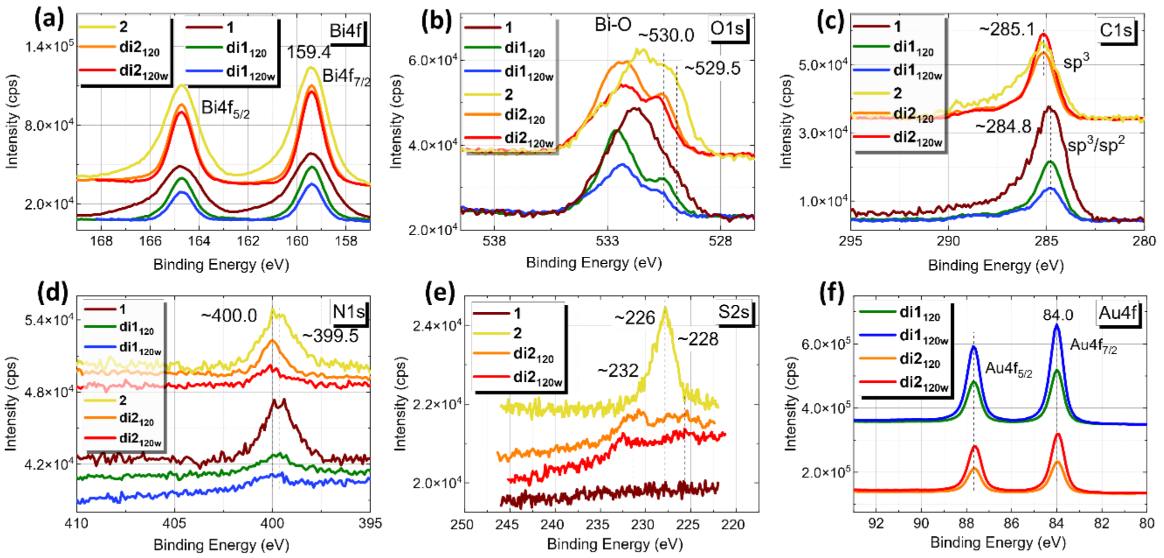

Figure S 13. XPS high resolution spectra of (a) Bi 4f, (b) O 1s, (c) C 1s, (d) N 1s, (e) S 2s, and (f) Au 4f for BiO-NC powders **1** and **2** as well as films **di1**$_{120}$, **di1**$_{120w}$, **di2**$_{120}$, and **di2**$_{120w}$. The systematic shift of about 0.5 eV toward lower binding energies in the O 1s, N 1s (and maybe S 2s) spectra for powders **1** and **2** can be due to an uncompensated charging effect or degradation of the sample. A significant broadening of the core level peaks is another evidence of the charging effect.

Table S 4. Sample compositions determined from the XPS high-resolution spectra (cf. Figure S12.). The O$_{Bi-O}$ and O$_{organic}$ contributions were obtained by fitting of the O 1s spectra similar to that in Figure 2 in the main text. The composition of the powder sample is in good agreement with the calculated formula. The overestimated oxygen and carbon contents in films di2$_{120}$ and di2$_{120w}$ are mostly due to the adventitious carbon. Note that the Bi:O:S:N element ratios of the films di2$_{120}$ and di2$_{120w}$ are close to each other, indicating integrity of the already deposited film after rinsing with ethanol.

	Calculated formula	Calculated ratio	XPS of cluster **2** (powder)	XPS of **di2**$_{120}$ (film)	XPS of **di2**$_{120w}$ (film)	Calculated	XPS of cluster **2** (powder)	XPS of **di2**$_{120}$ (film)	XPS of **di2**$_{120w}$ (film)
		Atomic %				Elemental ratios normalized to Bi content			
Bi	38	8.14	8.20	5.71	5.04	1	1	1	1
O$_{Bi-O}$	45	9.64	9.09	8.80	7.91	1.18	1.11	1.54	1.57
O$_{organic}$	96	20.56	18.99	25.38	19.96	2.53	2.32	4.45	3.96
C	240	51.39	53.34	54.93	63.11	6.32	6.50	9.62	12.52
S	24	5.14	5.21	1.55	1.42	0.64	0.64	0.27	0.28
N	24	5.14	5.18	3.63	2.56	0.63	0.63	0.64	0.51

3.4 AFM results

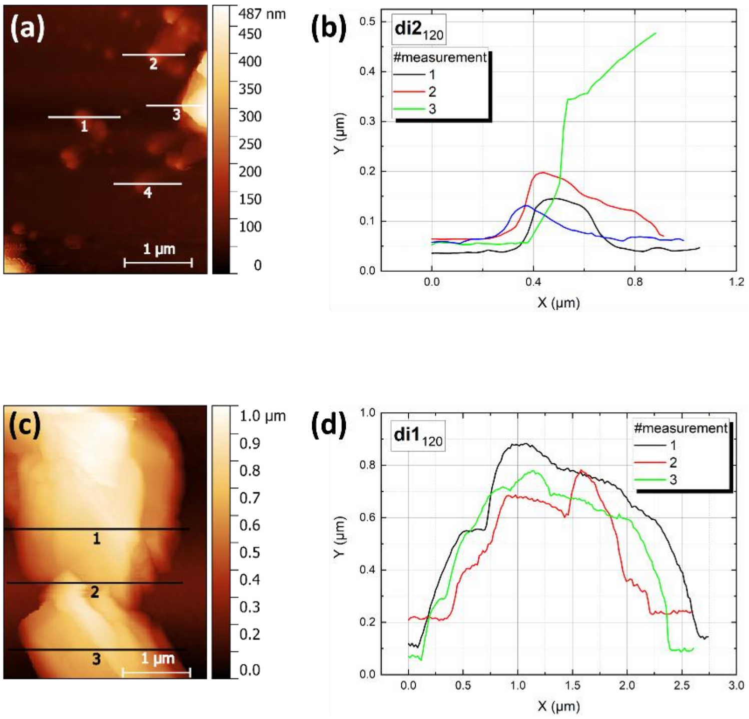

Figure S 14. (a) AFM image of BiO-NC **2** with profiles taken at four different spots across agglomerations of BiO-NC **2** (b). (c) cluster **1** agglomerations with profiles taken at three different spots demonstrated in (d).

Table S 5. Max. height, diameter, and area extracted from the AFM images and profiles from Figure S14.Figure S 14 **A significant height and area difference between BiO-NC 2 (di2$_{120}$) and BiO-NC 1 (di1$_{120}$) can be distinguished, where the BiO-NC 2 (di2$_{120}$) seem to agglomerate much less according to the strong interaction with the Au surfaces.**

Agglomeration	Max. Height	Diameter	Area
cluster **2** (**di2**$_{120}$)			
1	161.3 nm	445.1 nm	0.816 µm^2
2	161.8 nm	660.2 nm	0.897 µm^2
4	183.4 nm	640.3 nm	0.450 nm2
cluster **1** (**di1**$_{120}$)			
1	1.0 µm	2573.2 nm	6.723 µm^2
3	770.5 nm	1400.3 nm	-

3.5. SEM results

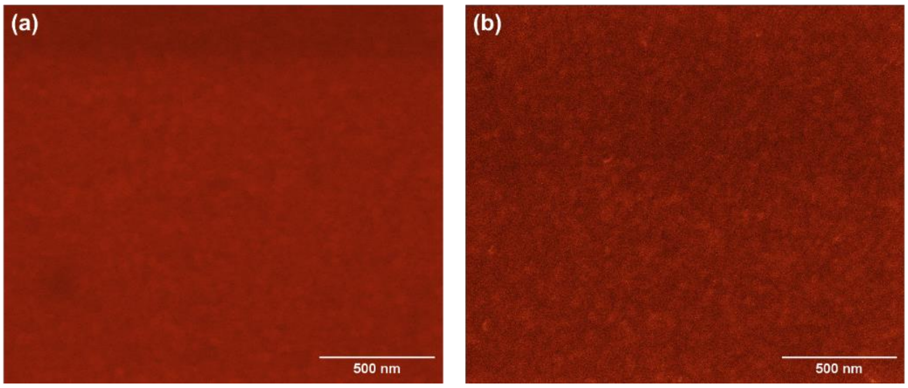

Figure S 15. SEM images for cluster **di1**$_{120w}$ (a) and **di2**$_{120w}$ (b).

3.6. Overview crystallographic density of different BiO-NCs

Table S 6. Overview of different BiO-NCs and their crystallographic density with the corresponding reference.

BiO-NCs	Density/ g·cm^{-3}	Reference
$[Bi_{38}O_{45}(OMc)_{24}(EtOH)_{13}]$	3.57	5
$[\{Bi_{38}O_{45}(O_2CC_6H_4\text{-}2\text{-}NH_2)_4(NO_3)_{20}(dmso)_{24}\}\text{-}$<br>$\{Bi_{38}O_{45}(O_2CC_6H_4\text{-}2\text{-}NH_2)_4(NO_3)_{20}(dmso)_{24}\}\cdot 6dmso]$	3.54	9
$[Bi_{38}O_{45}(HSal^4Me)_{24}(dmso)_{14}(H_2O)_2]\cdot 4H_2O$	2.87	10
$[Bi_{38}O_{45}(HSal)_{22}(OMc)_2(dmso)_{15}(H_2O)]\cdot dmso\cdot 2H_2O$	3.07	11
$[Bi_{38}O_{45}(HSal)_{22}(OH)_2(dmso)_{16.5}]\cdot dmso\cdot H_2O$	2.91	2

3.7. XPS layer thickness calculation

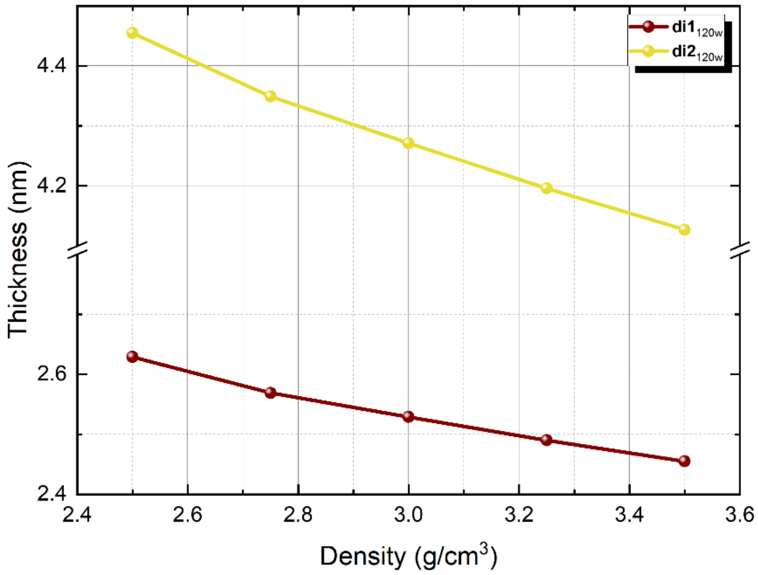

Figure S 16. XPS derived thickness of the films **di1**$_{120w}$ and **di2**$_{120w}$ calculated with different bulk density of clusters in the range between (2.5 – 3.5) g·cm^{-3} according to typical crystallographic densities of similar BiO-NCs. In the UHV the actual cluster density may deviate from the estimated crystallographic ones.

Table S 7. Thickness estimation of the adventitious carbon on bare Au substrates used for BiO-NCs deposition. Thickness is derived from the XPS method using formula (3) in the main text. Since adventitious carbon is not a defined material, the inelastic mean free path (λ) of diamond, graphite, and BiO nanoclusters 1 and 2 were taken for the calculations.

	peak name	peak KE (eV)	λ (nm)	peak area (a. u.)	d (nm)
diamond-like carbon	Au4f	1399	3.65	1	2.5 ± 0.5
	$Au4p_{3/2}$	938	2.69	0.78	
graphitic carbon	Au4f	1399	3.47	1	2.3 ± 0.5
	$Au4p_{3/2}$	938	2.55	0.78	
BiO-NC **2** in **di2**$_{120w}$	Au4f	1399	2.86	1	1.9 ± 0.5
	$Au4p_{3/2}$	938	2.10	0.78	
BiO-NC **1** in **di1**$_{120w}$	Au4f	1399	2.89	1	1.9 ± 0.5
	$Au4p_{3/2}$	938	2.12	0.78	

3.8. SE- fitting parameters for Arwin model

The Cauchy model is a dispersion layer and used, among others, to describe transparent thin films using a three-term equation, as shown in *Eq.* (1), where λ represents the wavelength in µm. The parameters A, B, and C are used to determine the refractive index dispersion [Complete Ease manual 6, pg.10-421][12]. *Eq.* (2) was used in addition to *Eq.* (1) to describe an Urbach absorption tail. Thereby k_{amp} and exp directly correspond to the extinction coefficient dispersion, where $E = h\frac{c}{\lambda}$, with c the velocity of light and h the Planck constant. The band edge is the onset of the absorption due to the band-gap between the HOMO and LUMO of the BiO-NC **2**.

$$n(\lambda) = A + \frac{B}{\lambda^2} + \frac{C}{\lambda^4} \quad (1)$$

$$k = k_{amp} e^{\exp(E - band\ edge)} \quad (2)$$

Table S 8. Description of Cauchy model parameters used for modelling the Cauchy layer thickness of BiO-NC 2 thin films on Au surfaces.

A = 1.45	B = 0.01 nm^{-2}	C = 0.00 nm^{-4}
k_{amp} = 0.00 (for E < band edge)	exp = 1.50	Band Edge = 3.50 eV

References

1. Morgenstern, A.; Thomas, R.; Sharma, A.; Weber, M.; Selyshchev, O.; Milekhin, I.; Dentel, D.; Gemming, S.; Tegenkamp, C.; Zahn, D. R. T.; Mehring, M.; Salvan, G., Deposition of Nanosized Amino Acid Functionalized Bismuth Oxido Clusters on Gold Surfaces. *Nanomaterials* **2022**, *12* (11), 1815., DOI: 10.3390/nano12111815
2. Mansfeld, D.; Miersch, L.; Rüffer, T.; Schaarschmidt, D.; Lang, H.; Böhle, T.; Troff, R. W.; Schalley, C. A.; Müller, J.; Mehring, M., From {$Bi_{22}O_{26}$} to Chiral Ligand-Protected {$Bi_{38}O_{45}$}-Based Bismuth Oxido Clusters. *Chem. Eur. J.* **2011**, *17* (52), 14805-14810., DOI: 10.1002/chem.201102437
3. Viezbicke, B. D.; Patel, S.; Davis, B. E.; Birnie III, D. P., Evaluation of the Tauc method for optical absorption edge determination: ZnO thin films as a model system. *Phys. status solidi b* **2015**, *252* (8), 1700-1710., DOI: 10.1002/pssb.201552007
4. Raju, G.; Ramesh, V.; Srinivas, R.; Sharma, G. V. M.; Shoban Babu, B., Differentiation of Boc-protected α,δ-/δ,α- and β,δ-/δ,β-hybrid peptide positional isomers by electrospray ionization tandem mass spectrometry. *J. Mass Spectrom.* **2010**, *45* (6), 651-663., DOI: 10.1002/jms.1756
5. Weber, M.; Thiele, G.; Dornsiepen, E.; Weimann, D. P.; Schalley, C. A.; Dehnen, S.; Mehring, M., Impact of the Exchange of the Coordinating Solvent Shell in [$Bi_{38}O_{45}(OMC)_{24}(dmso)_9$] by Alcohols: Crystal Structure, Gas Phase Stability, and Thermoanalysis. *Z. Anorg. Allg. Chem.* **2018**, *644* (24), 1796-1804., DOI: 10.1002/zaac.201800350
6. Weber, M.; Schlesinger, M.; Walther, M.; Zahn, D.; Schalley, C. A.; Mehring, M., Investigations on the growth of bismuth oxido clusters and the nucleation to give metastable bismuth oxide modifications. *Z. Kristallogr.—Cryst. Mater.* **2017**, *232* (1-3), 185-207., DOI: 10.1515/zkri-2016-1970
7. Miersch, L.; Schlesinger, M.; Troff, R. W.; Schalley, C. A.; Rüffer, T.; Lang, H.; Zahn, D.; Mehring, M., Hydrolysis of a Basic Bismuth Nitrate-Formation and Stability of Novel Bismuth Oxido Clusters. *Chem. Eur. J.* **2011**, *17* (25), 6985-6990, DOI: 10.1002/chem.201100673
8. Weber, M.; Rüffer, T.; Speck, F.; Göhler, F.; Weimann, D. P.; Schalley, C. A.; Seyller, T.; Lang, H.; Mehring, M., From a Cerium-Doped Polynuclear Bismuth Oxido Cluster to β-Bi_2O_3:Ce. *Inorg. Chem.* **2020**, *59* (6), 3353-3366., DOI: 10.1021/acs.inorgchem.9b03240
9. Wrobel, L.; Rüffer, T.; Korb, M.; Krautscheid, H.; Meyer, J.; Andrews, P. C.; Lang, H.; Mehring, M., Homo- and Heteroleptic Coordination Polymers and Oxido Clusters of Bismuth(III) Vinylsulfonates. *Chem. Eur. J.* **2018**, *24* (62), 16630-16644., DOI: 10.1002/chem.201803664
10. Schlesinger, M.; Pathak, A.; Richter, S.; Sattler, D.; Seifert, A.; Rüffer, T.; Andrews, P. C.; Schalley, C. A.; Lang, H.; Mehring, M., Salicylate-Functionalized Bismuth Oxido Clusters: Hydrolysis Processes and Microbiological Activity. *Eur. J. Inorg. Chem.* **2014**, *2014* (25), 4218-4227., DOI: 10.1002/ejic.201402493
11. Schlesinger, M.; Miersch, L.; Rüffer, T.; Lang, H.; Mehring, M., Two novel nanoscaled bismuth oxido clusters, [$Bi_{38}O_{45}(OMC)_{22}(C_8H_7SO_3)_2(DMSO)_6(H_2O)_{1.5}$]·2.5$H_2O$ and [$Bi_{38}O_{45}(HSal)_{22}(OMC)_2(DMSO)_{15}(H_2O)$]·DMSO·2$H_2O$. *Main Group Met. Chem.* **2013**, *36* (1-2), 11-19., DOI: 10.1515/mgmc-2012-0073
12. Sharma, A.; Matthes, P.; Soldatov, I.; Arekapudi, S. S. P. K.; Böhm, B.; Lindner, M.; Selyshchev, O.; Thi Ngoc Ha, N.; Mehring, M.; Tegenkamp, C.; Schulz, S. E.; Zahn, D. R. T.; Paltiel, Y.; Hellwig, O.; Salvan, G., Control of magneto-optical properties of cobalt-layers by adsorption of α-helical polyalanine self-assembled monolayers. *J. Mat. Chem. C* **2020**, *8* (34), 11822-11829., DOI: 10.1039/D0TC02734K
